# Harnessing Participatory Surveillance Cohorts and Proxy Indicators to Dynamically Track Epidemic Trends and Undiagnosed COVID-19 Infections in Singapore: Longitudinal Observational Study

**DOI:** 10.2196/85608

**Published:** 2026-06-10

**Authors:** Sheng En Alexius Matthias Soh, Aung Hein Aung, Wei Ling Brenda Ong, Kangwei Zeng, Jean-Marc Chavatte, Lin Cui, Raymond Valentine Tzer Pin Lin, Vanessa W Lim, May O Lwin, I-Cheng Mark Chen

**Affiliations:** 1 NCID Research Office, National Centre for Infectious Diseases, Singapore, Singapore; 2 Department of Epidemiology and Preventive Medicine, Tan Tock Seng Hospital, 11 Jln Tan Tock Seng, Singapore, 308443, Singapore, 65 96506340; 3 Advanced Methods and Analytics, Communicable Diseases Agency, Singapore, Singapore; 4 National Public Health Laboratory, Communicable Diseases Agency, Singapore, Singapore; 5 Microbiology Division, Department of Laboratory Medicine, National University Hospital, Singapore, Singapore; 6 Wee Kim Wee School of Communication and Information, Nanyang Technological University, Singapore, Singapore

**Keywords:** COVID-19, SARS-CoV-2, COVID-19 testing, public health surveillance, wastewater-based epidemiological monitoring, incidence, seroepidemiologic studies, cohort studies, logistic models, multilevel analysis

## Abstract

**Background:**

Accurate COVID-19 incidence estimates, including undiagnosed cases, are vital for epidemic management but are often unavailable in real time. Participatory surveillance can capture community illness episodes; however, quantifying undiagnosed infections remains difficult. We assessed a Singaporean cohort to estimate medically unattended COVID-19 infections by combining symptom models with proxy epidemic indicators.

**Objective:**

This study aims to estimate COVID-19 incidence and medically attended fractions using participatory surveillance data and to evaluate the consistency of these estimates against independently derived serological measures of infection in a community cohort in Singapore.

**Methods:**

We analyzed 11 survey waves (September 2021 to November 2022) from the SOCRATEs (Strengthening Our Community’s Resilience Against Threats from Emerging Infections) community cohort (n=1899), spanning Delta and Omicron variant waves. Respondents reported recent illness, symptoms, health care use, and COVID-19 diagnoses. Multilevel logistic regression of medically attended episodes estimated the probability of COVID-19 in unattended episodes, incorporating symptoms and external indicators—wastewater viral-load index and health care staff surveillance. The estimates of total infections and medically attended fractions were validated against independent serological survey results.

**Results:**

Among 2284 illness episodes, 756 were diagnosed with COVID-19, of which 62.4% (472/756) were medically attended. Health care–seeking declined from 83.9% (26/31) of COVID-19 episodes early in 2022 to 55.3% (47/85) by late 2022. Regression models demonstrated strong associations between COVID-19 infection and key symptoms and epidemic activity indicators. Estimated total infections were substantially higher than notified cases, reaching 1.0 to 2.9 times the reported incidence across successive variant waves. Model-based estimates of medically attended fractions were broadly consistent with serological benchmarks. Incidence estimates closely matched serological estimates in earlier intervals, with slight overestimation in later periods due to reinfection.

**Conclusions:**

Participatory surveillance, when combined with probabilistic modeling and external indicators of epidemic activity, can generate robust estimates of infection incidence and health care use. Agreement with serological data supports the validity of this integrated framework, although discrepancies persist in later epidemic phases due to reinfection dynamics. This approach provides a scalable and timely complement to traditional and serological surveillance systems.

## Introduction

The COVID-19 pandemic disrupted global public health systems in unprecedented and profound ways between 2020 and 2023. With its high transmissibility, overlap in clinical manifestations with endemic respiratory infections, potential for severe health outcomes, and capacity for asymptomatic transmission, COVID-19 rapidly evolved into a worldwide health crisis. Traditional epidemiological surveillance mechanisms, fundamental to policy decisions and epidemic response, encountered significant limitations as the pandemic rapidly progressed [1-4]. These included delays in data collection, analysis, and dissemination. To inform policymakers about preventable mortality and the impact of an infection on health systems, and hence the necessity of containment measures, an appropriate assessment of disease severity is paramount [5]. However, this relies critically on timely and accurate estimates of the proportion of the population that has been infected. Estimating the proportion infected is also critical for predicting an epidemic’s trajectory, assessing susceptibility to future waves of infection, and formulating vaccination strategies [6]. However, reliable data were not readily available during the early phases of the pandemic [7] and were largely unavailable in near real time. Serological studies [8,9] eventually provided clarity on the proportions infected, but they arrived too late to influence many critical decisions in earlier pandemic response efforts.

A potential method to address the gap is participatory surveillance [10-12], using digital technology [13-16] to collect near real-time [17,18] community data on illness and unreported cases. The participatory surveillance concept has been widely studied, particularly for influenza, with a 2023 review by Atkins et al [19] describing the advantages of such systems to complement traditional syndromic surveillance by providing almost real-time insights. Although work has compared trends from participatory surveillance [20-23] with data from national-level sources and laboratory-based surveillance [24-29], most of this work has focused on influenza and has not elaborated on how to integrate such data sources to estimate population-level incidence, prevalence, and attack rates for successive epidemic waves.

Interpreting infection prevalence remains challenging due to changing health-seeking behaviors [30] and overlapping symptoms of infections. A potential simplification is to assume episodes without health care visits have the same infection prevalence as outpatient cases, but this may be inaccurate. Our previous participatory surveillance study showed symptoms were associated with increased health care visits and likelihood of infection [31]. Recognizing this, a German study combined participatory and virological surveillance data while stratifying episodes by symptom profile [32]. The approach did yield higher incidence estimates than traditional systems, with a good temporal correlation with wastewater surveillance data. Innovations such as wastewater testing [33-36] and antigen rapid tests (ARTs) are now common [33-36] and can be timely epidemic indicators but cannot estimate infection rates. What is needed is a framework for combining these modalities with participatory cohorts to accurately estimate infection proportions and validate such approaches.

We address the above issues through a participatory surveillance cohort active in Singapore during the COVID-19 pandemic, which conducted repeated, digitally administered surveys to capture illness episodes, associated health care–seeking behaviors, and symptoms. Leveraging this, we aimed to do the following:

1. Evaluate the use of our participatory surveillance cohort for near real-time monitoring of COVID-19 activity in Singapore.
2. Demonstrate an approach that integrates participatory surveillance with other data streams (eg, wastewater testing, health care staff surveillance, percentage positive for COVID-19 at sentinel clinics) to estimate the additional amount of COVID-19 that would not be medically attended and, hence, not notified as cases to the Ministry of Health.
3. Compare our findings against passive case notifications and results from population-based serological surveys to assess the validity of our approach.

In doing so, we articulate a framework to integrate participatory surveillance with other surveillance streams to produce near real-time estimates of incidence rates for diagnosed and undiagnosed COVID-19, which could also be applied to other infections.

## Methods

### Study Design

Details of the SOCRATEs (Strengthening Our Community’s Resilience Against Threats from Emerging Infections) study were previously described in Lim et al [37]. Participants provided consent either in-person or through teleconference platforms. Follow-ups were conducted through online surveys, with each survey open for approximately 2 to 3 weeks after its launch date. The interval between survey launch dates varied depending on the needs at different stages of the pandemic and ranged from 1 to 2 months during the period of interest.

### Surveys on COVID-19 Infection Symptoms, Health Care Visits, and COVID-19 Diagnoses

**Figure 1.**
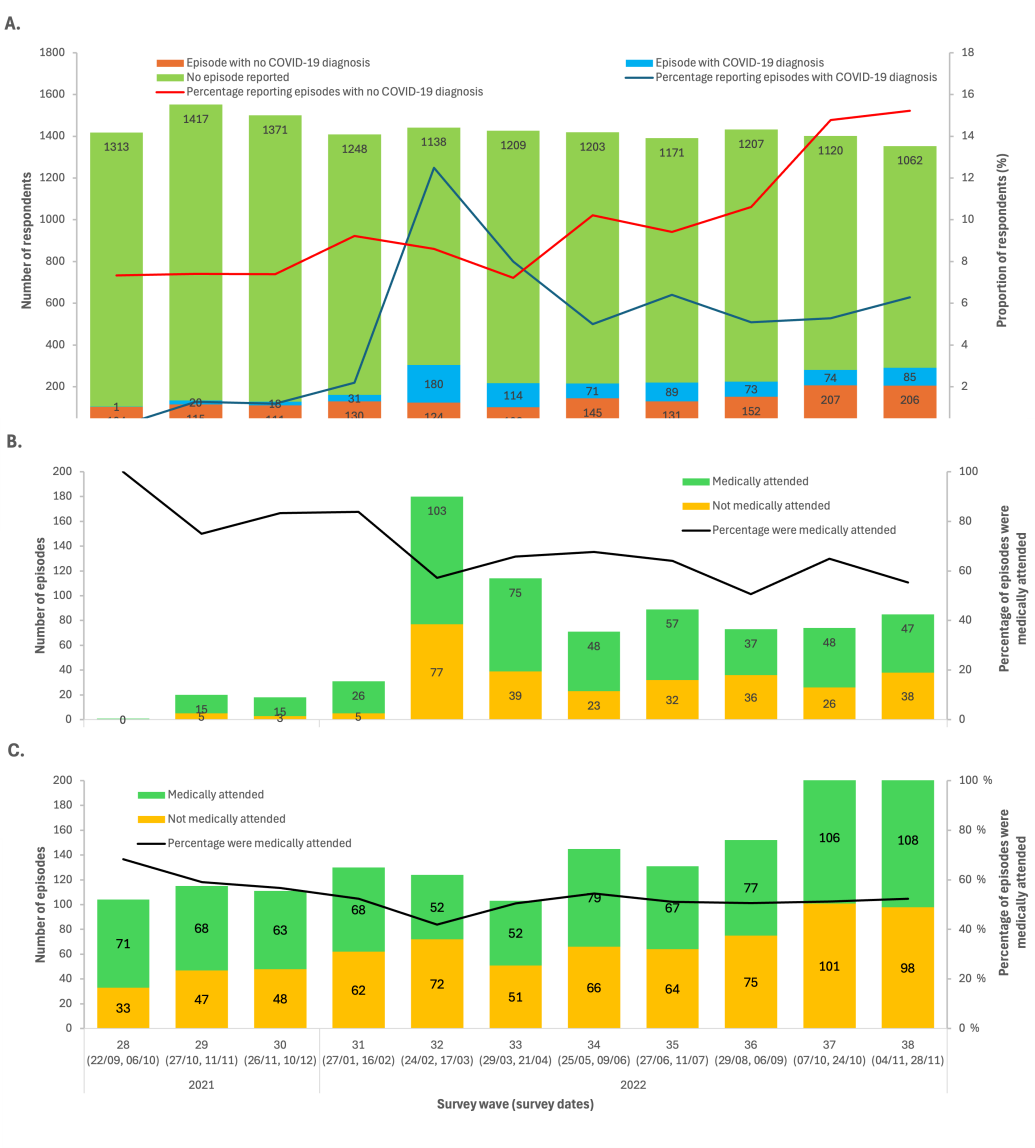
Proportion of episodes with and without COVID-19 diagnosis that were medically attended. (A) Total respondents, illness episodes, and COVID-19 diagnoses, (B) proportion of episodes with COVID-19 diagnoses that were medically attended, and (C) proportion of episodes with no COVID-19 diagnosis that were medically attended.

This analysis included 11 survey waves, spanning 4 epidemic peaks caused by the Delta COVID-19 variant, Omicron BA.1/BA.2, BA.5, and XBB subvariants (Figure 1A). The first survey (wave 28) opened on September 22, 2021, while the last survey (wave 38) closed on November 28, 2022. These waves included the period after COVID-19 ART self-test kits became widely available in mid-2021. They coincided with a transition in Singapore’s national approach to managing COVID-19 toward “living with COVID-19” [38,39]. This transition marked the end of comprehensive contact tracing and quarantine, and consequently, the transmission of COVID-19 was no longer confined to localized outbreaks. To support disease surveillance, all surveys during this period asked whether respondents had experienced acute illness episodes in the 28 days before submitting their response. If so, they were asked when the episode occurred, if their illness was accompanied by a core list of symptoms associated with COVID-19 (cough, runny nose, sore throat, breathlessness, loss of sense of smell, and feeling feverish), and whether they had a physical health care visit for their illness. Additional questions assessed whether the health care visit resulted in a diagnosis of COVID-19 (“medically diagnosed”) and whether they also self-tested positive by ART. Episodes that self-tested positive without a health care visit were considered “self-diagnosed.”

### Data Analysis

Our analysis aimed to estimate the incidence of COVID-19 infections, including those not medically attended and thus not reported to the Ministry of Health. Methodological details and notations used are provided in Table S1-1 in Multimedia Appendix 1. Briefly, the unit of analysis was the participant-response-day, with each participant contributing person-day observations to calculate incidence rates over the 28 days preceding each survey response. Episodes within this period contributed fully, while others were weighted for partial contribution (Tables S2 and S3 in Multimedia Appendix 1). In our analysis, we included all observed seroconversions within each interval and aligned the data by assuming an average lag of approximately 2 weeks [40,41] between infection onset and seroconversion. Syndromic incidence estimates are therefore aggregated over intervals shifted by 2 weeks relative to the corresponding serological sampling windows, defined using the median dates of the initial and terminal samples for each interval. For the estimation of ascertainment fractions, we computed this by including illness episodes reported up to 2 weeks prior to the initial sample of each interval as potentially contributing to observed seroconversions and classified these as medically attended where appropriate.

Time was not modeled using explicit calendar-date terms (eg, linear trends or interval-specific dummy variables). Instead, temporal variation in infection risk was incorporated through time-varying proxy indicators of epidemic activity, evaluated at the calendar dates corresponding to each participant-response-day observation. Depending on model specification, these indicators included the incidence of medically attended COVID-19 within the cohort, incidence among health care staff, wastewater viral-load index, or the proportion positive for COVID-19 at sentinel clinics. This approach allowed the model to capture nonlinear, wave-specific changes in transmission intensity without imposing parametric assumptions on calendar time.

To account for correlations arising from repeated observations, we fitted a multilevel logistic regression with random intercepts at 3 levels: participants with multiple responses, each representing observation time across dates. The models included demographics, symptoms, optional proxy indicators of epidemic activity—medically attended COVID-19 incidence, lab- and self-reported testing among health care staff [42], wastewater viral-load index (WVI) from the National Environment Agency [35], and the proportion positive for COVID-19 at sentinel clinics tested by the National Public Health Laboratory [43]. We did not impose an explicit temporal autocorrelation structure at the day level (eg, autoregressive terms), as survey responses were irregularly spaced in time, and epidemic-level temporal dependence was already represented by the external proxy indicators. All models were therefore interpreted as estimating symptom-infection relationships conditional on contemporaneous epidemic intensity rather than modeling time as an independent covariate.

During the study, the Ministry of Health [44,45] stipulated COVID-19 testing for all adults with respiratory symptoms presenting at health care facilities. We considered COVID-19 status to be accurate for medically attended episodes but less reliable for unattended episodes due to incomplete testing and lower self-testing accuracy. Therefore, only medically attended episodes were used in multivariable models to assess the predictive value of symptoms. These models were then used to predict the likelihood of COVID-19 in unattended episodes based on symptoms and epidemic timing. Since symptoms were crucial, analyses used only episodes with at least 1 of 6 symptoms: cough, runny nose, sore throat, breathlessness, loss of smell, or feeling feverish.

For a given calendar date, the logistic model’s predictions were used to estimate the average weighted probability that an illness episode could be COVID-19. This was then applied in 2 methods to estimate the proportion of COVID-19 cases that were medically attended. Both methods involved the sum of medically attended COVID-19 observations as the numerator and 1 part of the denominator. Method 1 included only 1 additional component in the denominator: the sum of observations for unattended episodes weighted by the probability of being COVID-19 over time. This approach ignores reports of self-diagnosed COVID-19 episodes. Conversely, method 2 incorporated into its denominator the total number of observations for both medically attended and self-diagnosed COVID-19 episodes, along with unattended episodes without a COVID-19 diagnosis, weighted by the corresponding probability of COVID-19.

The medically attended fraction of COVID-19 was stratified into 3 intervals guided by the collection dates for serological samples [46] against which the results were compared (Table S4 in Multimedia Appendix 1). These corresponded approximately to periods when the Delta, Omicron BA.1/BA.2, and Omicron BA.5/XBB variants were circulating. We also compared the cumulative incidence rates estimated from seroconversions, which approximate population-level infections when new infections still vastly outnumber reinfections (Multimedia Appendix 1).

The Institutional Review Board of the National Healthcare Group approved the SOCRATEs (reference 2018/01203, approved February 22, 2019) and serological (reference 2020/00805, approved July 23, 2020) studies. All statistical analyses were performed using STATA 18 on Windows 10, and *P* values <.05 were considered statistically significant. The STROBE checklist (Checklist 1) [47] was used to guide our reporting of results.

### Ethical Considerations

The study was conducted according to the guidelines of the Declaration of Helsinki and approved by the Institutional Review Board of the National Healthcare Group (study reference number 2018/01203 and study date of approval February 22, 2019). All participants involved in the study provided informed consent. The data were anonymized and deidentified prior to analysis. This is not a clinical trial.

## Results

Of the 2141 total participants in the SOCRATEs cohort (Table 1), 1899 responded to one or more of the survey waves (waves 28‐38). Of these, 159 (8%) were from door-to-door recruitment, 730 (38%) from other cohorts, and 1010 (53%) from referrals. Respondents for the included survey waves were similar to the overall cohort recruited and distributed evenly across age groups. The majority identified as Chinese (n=1687, 89%), held tertiary education (n=1096, 58%), were female (n=1151, 61%), and resided in 4‐ to 5-bedroom publicly owned flats (n=1097, 58%). Minority ethnic groups were underrepresented relative to population data from the Singapore Department of Statistics, as were individuals with fewer years of education (n=260, 14% with “O”/“N” level and below among respondents vs n=1,142,800, 37% in national data).

**Table 1.** SOCRATEs (Strengthening Our Community’s Resilience Against Threats from Emerging Infections) cohort demographics.

Demographics	SOCRATEs cohort as at December 31, 2022 (N=2141), n (%)	Participants with at least 1 survey after wave 28 (N=1899), n (%)	Population data (year 2023)^a^ (%)
Age (y)			
18‐29	378 (18)	328 (17)	20
30‐39	457 (21)	390 (21)	18
40‐49	403 (19)	362 (19)	18
50‐59	396 (18)	373 (20)	17
>60	507 (24)	446 (23)	28
Gender			
Male	866 (40)	748 (39)	49
Female	1275 (60)	1151 (61)	51
Ethnicity			
Chinese	1862 (87)	1687 (89)	74
Malay	72 (3)	47 (2)	14
Indian	153 (7)	119 (6)	9
Others	54 (3)	46 (2)	3
Education^b^			
“O” or “N” level and below	314 (15)	260 (14)	37
“A” level or polytechnic diploma	626 (29)	543 (29)	27
University or postgraduate	1201 (56)	1096 (58)	36
Housing type			
Publicly owned flat with ≤3 rooms	297 (14)	253 (13)	24
Publicly owned flat with 4‐5 rooms	1254 (59)	1097 (58)	54
Privately owned property	590 (28)	549 (29)	22
Pre-existing conditions			
Diabetes	98 (5)	84 (4)	—^c^
Hypertension	214 (10)	186 (10)	—

a Taken from singstat.gov.sg (figures may not add up to the totals due to rounding).

b Population education data restricted to 25 years and older because that is what is available.

c Not available.

Each wave included 1300 to 1600 respondents (Figure 1A) and reported 2511 illness episodes between September 22, 2021, and November 28, 2022. After excluding 227 episodes that occurred more than 28 days before the survey date, 2284 episodes were analyzed. Except for 2 brief gaps in December 2021 and July 2022, almost all dates within the study period fell within the survey response windows or were covered by the 28-day lookback period from the start of each window. The proportions reporting illness episodes increased from 7.4% in wave 28 to more than 20% by wave 38. Episodes with COVID-19 diagnoses increased from 1 (0.1%) episode in wave 28 to a peak of 180 (12.5%) in wave 32 and then ranged between 5% and 8% of responses from wave 33 onward. Episodes without a COVID-19 diagnosis ranged from 7% to 9% during waves 28 to 33 and then more than doubled to 15.2% by wave 38. Among episodes with a COVID-19 diagnosis (Figure 1B), 62.4% (472/756) were medically attended. This proportion decreased from about 80% in waves up to early 2022 to less than 60% by wave 38. In episodes without a COVID-19 diagnosis (Figure 1C), 811 out of 1528 (53.1%) were medically attended, and the proportion decreased from 68.3% in wave 28% to 52.4% by wave 38.

Among episodes with at least 1 of the 6 core symptoms, medically attended and unattended episodes had a median of 3 (IQR 2-4) versus 2 (IQR 1-4) symptoms respectively (*P*<.001), and all symptoms except runny nose were significantly more common (Table 2). However, most differences were less marked in stratified analysis by COVID-19 status. In COVID-19 episodes, the median number of symptoms was 4 (IQR 3-5) in both attended and unattended (IQR 3-4) episodes (*P*=.12). Symptom profiles were also similar. For instance, 88.4% versus 83.7% had cough (*P*=.09), and 91.8% versus 89.0% had sore throat (*P*=.24) in attended and unattended episodes, respectively. Not unexpectedly, attended episodes had a lower proportion of unreported temperatures (31.7% vs 49.7%; *P*<.001), but the distribution of temperature readings was not significantly different (*P*=.86). However, in episodes with no COVID-19 diagnosis, several symptoms were more common in medically attended versus unattended episodes, including cough (54.3% vs 42.4%; *P*<.001), shortness of breath (14.4% vs 11.7%; *P*=.07), and feeling feverish (45.9% vs 39.3%; *P*=.02). When comparing episodes with and without a COVID-19 diagnosis, the median number of symptoms was significantly greater, and all symptoms were significantly more common, both for medically attended and unattended episodes. COVID-19 episodes were also significantly more likely to report temperatures of 38.5 °C or higher. However, temperature readings were unavailable for a large proportion of observations and were therefore not used in multivariable analysis to discriminate COVID-19 from other illness episodes.

**Table 2.** Comparison of symptoms by COVID-19 diagnosis and whether the episode is medically attended or unattended.

Characteristics	All episodes with symptoms reported			Had COVID-19 diagnosis			No COVID-19 diagnosis			Comparison by COVID-19 diagnosis	
	A. Medically attended	B. Medically unattended	*P* value, A vs B	C. Medically attended	D. Medically unattended	*P* value, C vs D	E. Medically attended	F. Medically unattended	*P* value, E vs F	*P* value, C vs E	*P* value, D vs F
Number of episodes	1004	791	—^a^	464	209	—	540	582	—	—	—
Symptoms, median (IQR)	3 (2-4)	2 (1-4)	<.001^b^	4 (3-5)	4 (3-4)	.12^b^	2 (1-3)	2 (1-3)	.002^b^	<.001^b^	<.001^b^
Reported symptoms, n (%)											
Cough	703 (70)	422 (53.4)	<.001	410 (88.4)	175 (83.7)	.09	293 (54.3)	247 (42.4)	<.001	<.001	<.001
Runny nose	676 (67.3)	516 (65.2)	.35	364 (78.4)	157 (75.1)	.34	312 (57.8)	359 (61.7)	.18	<.001	<.001
Sore throat	781 (77.8)	544 (68.8)	<.001	426 (91.8)	186 (89)	.24	355 (65.7)	358 (61.5)	.14	<.001	<.001
Shortness of breath	195 (19.4)	111 (14)	.003	112 (24.1)	43 (20.6)	.31	83 (15.4)	68 (11.7)	.07	<.001	.002
Loss of smell	119 (11.9)	63 (8)	.007	92 (19.8)	35 (16.7)	.35	27 (5)	28 (4.8)	.88	<.001	<.001
Felt feverish	579 (57.7)	376 (47.5)	<.001	331 (71.3)	147 (70.3)	.79	248 (45.9)	229 (39.3)	.03	<.001	<.001
Felt feverish but no temperature reported^c^, n (%)	225 (38.9)	214 (56.9)	<.001	105 (31.7)	73 (49.7)	<.001	120 (48.4)	141 (61.6)	.004	<.001	.02
Reported temperature (℃) in those felt feverish^d^, n (%)			.06			.86			.11	.001	<.001
<38.0	155 (43.7)	79 (54.1)		84 (37.2)	26 (40)		71 (55)	53 (65.4)			
38.0-38.4	81 (22.8)	32 (21.9)		52 (23)	13 (20)		29 (22.5)	19 (23.5)			
>38.5	119 (33.5)	35 (24)		90 (39.8)	26 (40)		29 (22.5)	9 (11.1)			

a Not applicable.

b Wilcoxon rank-sum test; all other *P* values by chi-squared tests

c Denominator is those who felt feverish.

d Denominator is those who felt feverish and reported a valid temperature.

Table 3 shows the regression model results for 1004 medically attended episodes from 705 participants. In univariable analyses, those aged 30 to 39 years had lower odds of COVID-19 than those younger than 30 years, but there were no other significant associations with sociodemographic variables or pre-existing medical conditions. In contrast, significant positive associations (at *P*<.01) were observed for all symptoms and indicators of epidemic activity in univariable analysis. Across all multivariable models, significant associations remained for 4 symptoms and all indicators of epidemic activity in the respective models. For instance, when using WVI as a proxy indicator, the adjusted odds ratios (95% CIs) for cough, sore throat, loss of sense of smell, and feeling feverish were 3.6 (2.2‐5.7), 3.8 (2.2‐6.5), 4.3 (2.2‐8.4), and 2.8 (1.9‐4.1), respectively, while the log of WVI had an adjusted odds ratio of 2.2 (1.8‐2.8).

**Table 3.** Univariable and multivariable logistic regression showing results for demographics, symptoms, proxy indicators and random intercept terms.

Characteristics	Univariable analyis, OR (95% CI)	Multivariable analysis^a^ for different proxy indicator options			
		No proxy indicators, OR (95% CI)	Log of incidence per 1000 person days, OR (95% CI)^b^	Log of incidence per 1000 health care staff days, OR (95% CI)	Log of wastewater viral-load index, OR (95% CI)
Age (y; vs 18‐29)					
30‐39	0.58 (0.37‐0.91)^c^	0.49 (0.25‐0.94)^c^	0.51 (0.26‐1)	0.54 (0.28‐1.04)	0.55 (0.29‐1.04)
40‐49	1.03 (0.65‐1.63)	0.83 (0.43‐1.6)	0.8 (0.41‐1.57)	0.83 (0.43‐1.62)	0.86 (0.46‐1.64)
50‐59	0.97 (0.6‐1.58)	0.91 (0.46‐1.81)	0.95 (0.47‐1.92)	0.98 (0.49‐1.97)	1.01 (0.52‐1.97)
>60	1.56 (0.93‐2.63)	1.57 (0.71‐3.46)	1.43 (0.64‐3.23)	1.48 (0.67‐3.29)	1.56 (0.72‐3.36)
Female gender (vs male)	0.77 (0.58‐1.03)	0.79 (0.55‐1.14)	0.73 (0.51‐1.07)	0.74 (0.51‐1.07)	0.73 (0.51‐1.05)
Race (vs Chinese)					
Malay	0.81 (0.39‐1.69)	0.92 (0.4‐2.13)	1.07 (0.4‐2.89)	1.13 (0.43‐3)	1.06 (0.42‐2.71)
Indian	0.74 (0.42‐1.31)	0.65 (0.28‐1.52)	0.63 (0.26‐1.49)	0.63 (0.27‐1.5)	0.64 (0.28‐1.47)
Others	0.53 (0.2‐1.38)	0.56 (0.15‐2.17)	0.49 (0.13‐1.87)	0.49 (0.13‐1.84)	0.48 (0.13‐1.8)
Housing type (vs publicly owned flat with ≤3 rooms)					
Publicly owned flat with 4‐5 rooms	0.81 (0.51‐1.28)	0.89 (0.49‐1.63)	0.87 (0.46‐1.62)	0.86 (0.47‐1.59)	0.87 (0.48‐1.57)
Privately owned property	1.13 (0.67‐1.91)	1.13 (0.57‐2.25)	1.14 (0.56‐2.31)	1.13 (0.57‐2.26)	1.13 (0.58‐2.22)
Education (vs ’'O” or “N” level & below)					
“A” level or polytechnic diploma	0.69 (0.41‐1.16)	0.82 (0.4‐1.7)	0.85 (0.4‐1.79)	0.89 (0.42‐1.85)	0.91 (0.44‐1.89)
University or postgraduate	0.66 (0.41‐1.06)	0.74 (0.37‐1.48)	0.81 (0.39‐1.67)	0.82 (0.4‐1.66)	0.85 (0.42‐1.71)
Occupational status (vs employed)					
Student	0.99 (0.55‐1.78)	0.58 (0.22‐1.51)	0.63 (0.23‐1.69)	0.61 (0.23‐1.61)	0.64 (0.26‐1.63)
Self-employed	1.15 (0.66‐2.01)	1.4 (0.76‐2.6)	1.35 (0.69‐2.62)	1.43 (0.75‐2.75)	1.32 (0.71‐2.47)
Not employed nor studying	1.44 (0.93‐2.22)	1.24 (0.68‐2.23)	1.21 (0.66‐2.22)	1.24 (0.69‐2.26)	1.19 (0.67‐2.13)
Pre-existing conditions (vs none)					
Diabetes	1.21 (0.55‐2.65)	1.21 (0.44‐3.35)	1.16 (0.44‐3.05)	1.2 (0.45‐3.21)	1.24 (0.47‐3.24)
Hypertension	1.36 (0.84‐2.21)	0.8 (0.41‐1.57)	0.76 (0.39‐1.46)	0.78 (0.41‐1.51)	0.75 (0.39‐1.43)
Symptoms					
cough	7.38 (4.88‐11.16)^e^	4.24 (2.62‐6.87)^e^	3.56 (2.17‐5.82)^e^	3.35 (2.06‐5.44)^e^	3.57 (2.23‐5.71)^e^
runny nose	3 (2.13‐4.25)^e^	1.42 (0.95‐2.12)	1.42 (0.94‐2.15)	1.4 (0.93‐2.11)	1.38 (0.93‐2.05)
sore throat	6.96 (4.35‐11.15)^e^	4.24 (2.44‐7.39)^e^	3.69 (2.13‐6.39)^e^	3.81 (2.21‐6.57)^e^	3.78 (2.21‐6.46)^e^
breathlessness	1.95 (1.33‐2.87)^d^	1.25 (0.78‐1.99)	1.21 (0.75‐1.95)	1.17 (0.73‐1.88)	1.18 (0.74‐1.88)
loss of smell	6.23 (3.56‐10.89)^e^	3.89 (2.01‐7.52)^e^	5.18 (2.49‐10.77)^e^	5.25 (2.52‐10.94)^e^	4.31 (2.21‐8.38)^e^
feeling feverish	3.45 (2.41‐4.94)^e^	3.24 (2.18‐4.81)^e^	2.9 (1.96‐4.3)^e^	2.87 (1.94‐4.25)^e^	2.83 (1.93‐4.13)^e^
Proxy indicators, log of					
Incidence per 1000 person days	3.37 (2.6‐4.35)^e^	^-f^	3.11 (2.33‐4.15)^e^	^-f^	^-f^
Incidence per 1000 health care staff days	2.58 (2.09‐3.19)^e^	^-f^	^-f^	2.32 (1.84‐2.94)^e^	^-f^
Wastewater viral-load Index	2.5 (2.07‐3.02)^e^	^-f^	^-f^	^-f^	2.22 (1.79‐2.75)^e^
Random intercept terms					
Between participants	^-f^	0.55 (0.13‐2.33)^e^	0.68 (0.18‐2.54)^e^	0.69 (0.19‐2.44)^e^	0.63 (0.17‐2.32)^e^
Between responses within particpants	^-f^	0 (0‐0)^e^	0 (0‐0)^e^	0 (0‐0)^e^	0 (0‐0)^e^

a Full model includes symptoms, respective options for proxy indicators shown in the table, plus demographics and random intercept terms for variability between participants and between survey responses within participants.

b From medically ascertained episodes within SOCRATEs cohort.

c *P*<.05.

d *p < .01*

e *P*<.001.

f Not applicable

**Figure 2.**
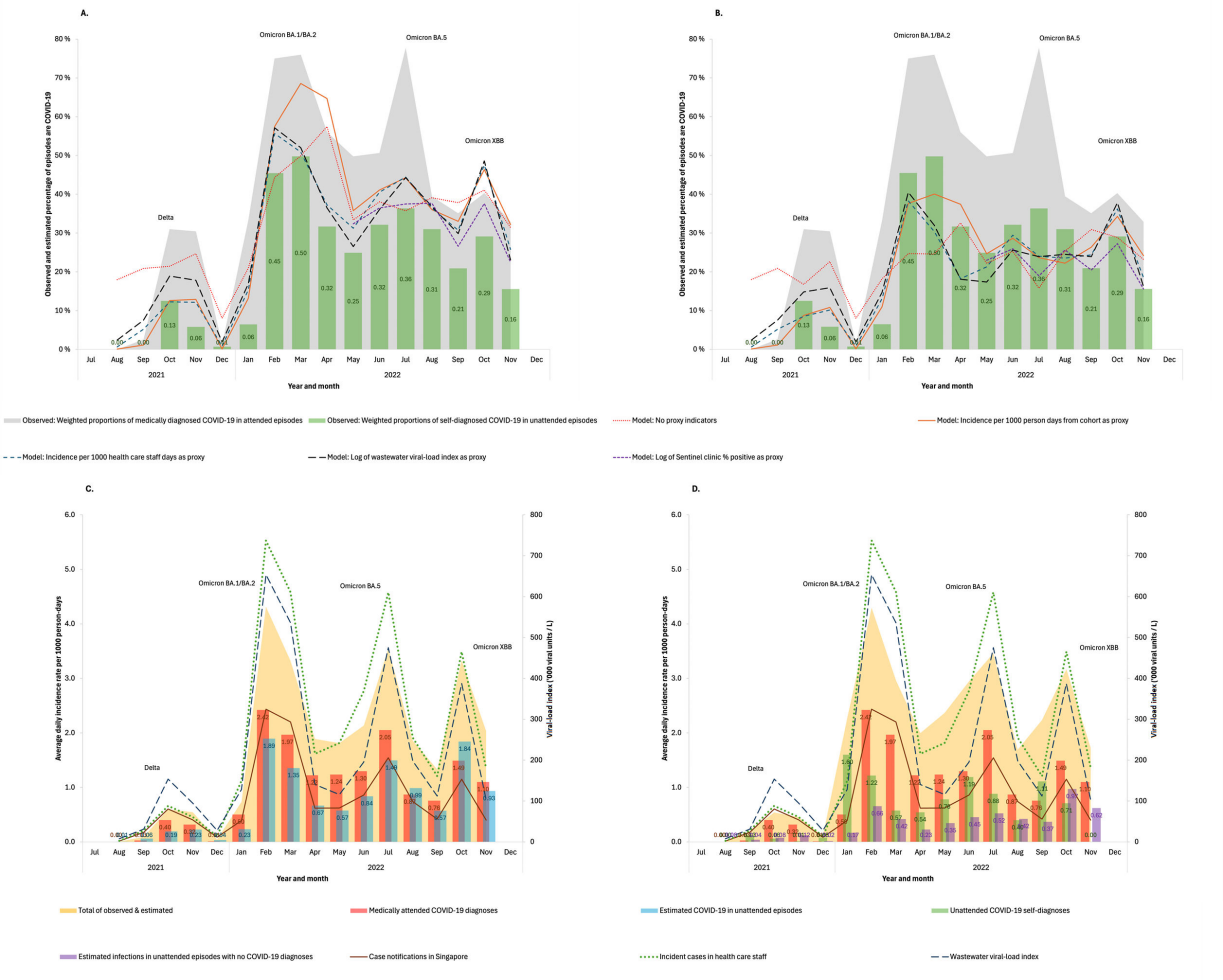
Model estimates of proportion infected and the incidence rates from estimating infections for unattended episodes with and without COVID-19 diagnosis. (A) Model estimates of proportion infected for unattended episodes, (B) model estimates of proportion infected for unattended episodes with no COVID-19 diagnosis, (C) method 1: incidence rates from estimating infections in unattended episodes (using wastewater viral-load index [WVI] as proxy), and (D) method 2: incidence rates from estimating infections in unattended episodes with no COVID-19 diagnosis (using WVI as proxy).

Key epidemics of COVID-19 were discernible as peaks in the weighted proportions diagnosed with COVID-19 for both medically attended [48] (gray area) and unattended episodes (green bars), but were clearer in the former. When any indicator of epidemic activity was included, the regression models gave similar trends in the estimated proportions infected both for all unattended (Figure 2A) and unattended episodes with no COVID-19 diagnosis (Figure 2B), even when using sentinel clinic percentage positive where data were available only from May 2022 onward. Trends in the model without any proxy indicator were much less discernible. The differential prevalence of key symptoms (Table 2) led to estimated infections for unattended episodes being generally lower than the observed weighted proportions infected for medically attended episodes (Figure 2A). Among unattended episodes, estimated infections were marginally higher than weighted proportions of self-diagnosed COVID-19, particularly during epidemic peaks (Figure 2A). Conversely, estimated infections among unattended episodes with NO COVID-19 diagnosis (Figure 2B) were mostly lower than the observed weighted proportions for self-diagnosed COVID-19.

Because WVI time-series data were the most complete, we used them to model COVID-19 incidence for unattended episodes in Figure 2C and D. There was a slight divergence in the latter half of the study period, but nationally notified cases and medically attended COVID-19 infections observed in the cohort were very similar, with incidence rates of 2.4 per 1000 persons per day at the peak of Omicron BA.1/BA.2 activity in February 2022. However, the incidence of infections was likely much higher, particularly at later time points, as a substantial proportion of unattended episodes were likely COVID-19 infections. Adding unnotified infections estimated from method 1 (yellow area), total infections were 1.0, 1.8, 2.3, and 2.9 times the rate of notified cases during the peaks of the Delta, Omicron BA.1/BA.2, Omicron BA.5, and Omicron XBB epidemics, respectively (Figure 2C). Total infections from method 1 were nearly equivalent to those from method 2, which separately estimated undiagnosed COVID-19 in unattended episodes with no COVID-19 diagnosis and summed this with incidence rates of self-diagnosed COVID-19. Although estimated by fitting to WVI data, total infection rates showed strong temporal correlation and were numerically close to those from health care staff.

**Figure 3.**
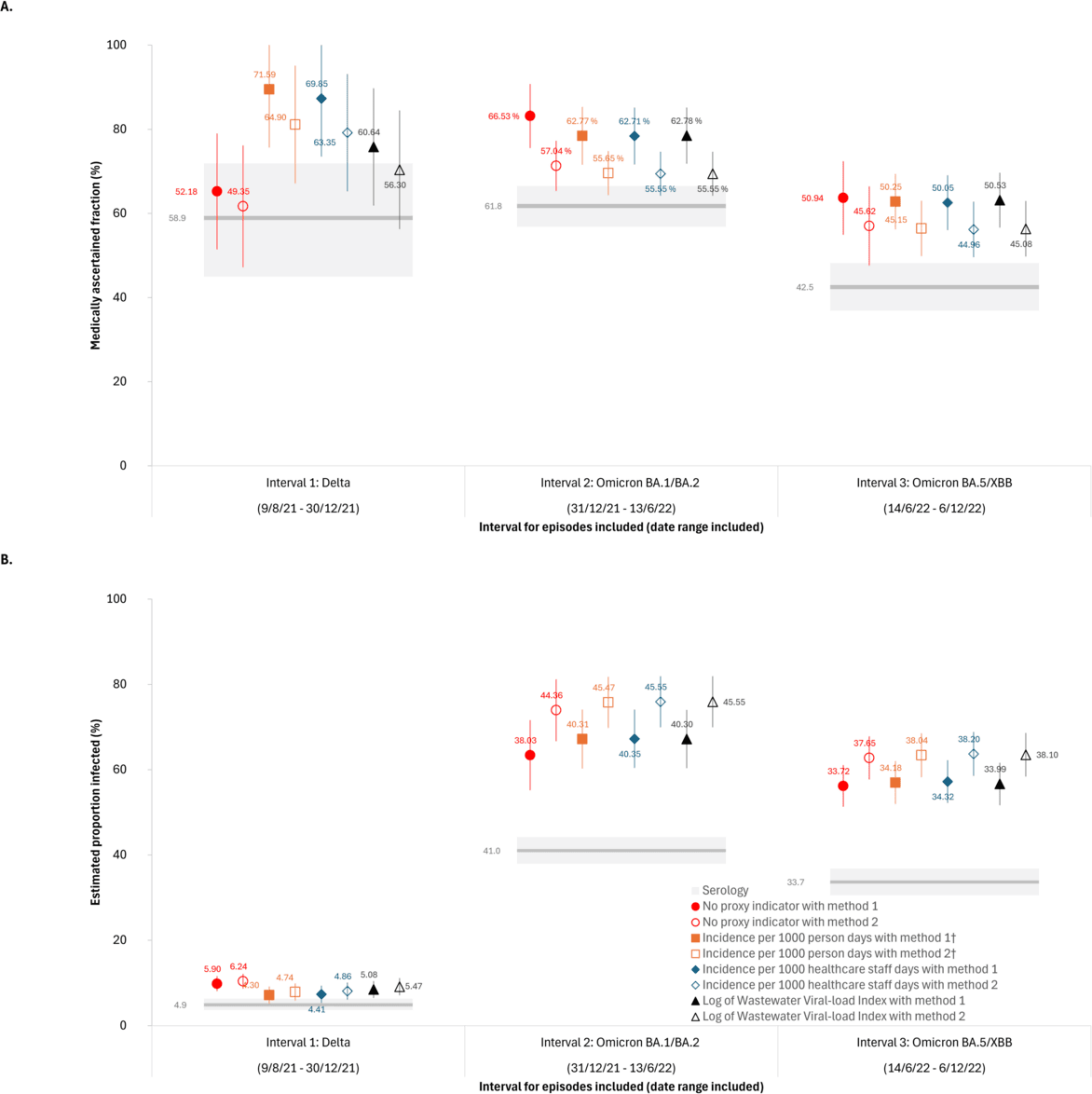
Estimates of medically ascertained fractions and attack rates as compared to those from serology. (A) Estimates of medically ascertained fractions compared with those from serology and (B) estimates of attack rates compared with those from serology.

Figure 3A shows that, across the first, second, and third intervals, respectively, 58.9%, 61.8%, and 42.5% of serologically detected incident COVID-19 infections were medically attended. For the proxy indicators, we omitted the sentinel clinic percentage positive because insufficient data were available for the first 2 intervals. However, models using the other 3 indicators gave fairly similar results (<20% difference), other than for the first interval, where estimates ranged from ~56% to~71%. For the latter 2 intervals, estimates for medically attended fractions deviated from those by serology by <9%. Estimates for the last interval were 3% to 9% higher but had bootstrapped CIs overlapping with the corresponding results from serology. For methods 1 and 2, the total deviance from serology over all 3 intervals was the smallest using WVI and staff surveillance, respectively.

Based on the cumulative incidence of seroconversions divided by the number of valid samples, the serological study estimated that 4.9% were infected in interval 1% and 41% in interval 2 (Figure 3B). The 3 proxy indicators for which data were available gave similar estimates of 4.4% to 5.5% for interval 1% and 40.3% to 45.6% for interval 2. For interval 3, estimates from the 3 indicators ranged between 34% and 38.2%. This was generally higher than the 33.7% from serology, which could not estimate additional infections among those who were already seropositive due to past infections, and the final samples were collected about 1 month after our participatory surveillance data ended.

## Discussion

The usefulness of digitally administered participatory surveillance for enhancing situational awareness during public health emergencies has been recognized [10,49-51] for several years. However, applying it to generate quantitative estimates of absolute incidence has remained difficult due to the lack of a known denominator and challenges in determining infection status among digitally reported cases. Our study makes progress in 2 areas. First, by integrating symptom follow-up questionnaires within a pre-enrolled, demographically characterized cohort, we were able to directly calculate person-time denominators rather than estimating them from user activity metrics. Second, by linking cohort data to another indicator of community transmission intensity, we could use regression techniques to estimate the likelihood of undiagnosed infections, thus overcoming the dependence between infection detection and incidence estimation.

Our analysis shows that a participatory syndromic surveillance cohort—when integrated analytically with an external, closely timed indicator of epidemic activity, such as health care staff incidence rates, WVI, or sentinel clinic test positivity (though we had limited data points for this)—can generate estimates of COVID-19 incidence that are both time-correlated and numerically similar to national notifications over approximately 18 months covering 4 distinct SARS-CoV-2 variant waves. Additionally, the multilevel regression indicated that the likelihood of having COVID-19 among surveyed illness episodes depended systematically on symptom profiles. Symptoms among COVID-19 episodes were strikingly similar between respondents who sought health care and those who did not. Conversely, non-COVID respiratory episodes showed a clear gradient of symptom severity, with medically attended episodes reporting a higher median number of symptoms and greater prevalence of certain symptoms (eg, cough, feeling feverish). These findings support earlier research suggesting that individuals’ decision to seek health care is more influenced by how severe they perceive their symptoms to be than by the illness’s cause alone [31]. By combining symptom data with proxy indicators of epidemic activity, we could then plausibly estimate undiagnosed infections in episodes that were not medically attended. External validation against a serological cohort on the proportions of COVID-19 cases that were medically attended and the proportion infected further reinforced the reliability of this method.

The increase in self-diagnosed, nonpresenting COVID-19 episodes and the decline in health care–seeking from February 2022 suggest the behavioral impact of widespread ART kits and changing risk perception. As the epidemic evolved, case notifications likely underestimated infections. However, a naive approach applying health care–based test-positivity to all reported episodes of acute respiratory illness in the community would likely overestimate infections, since milder illness is less likely to be COVID-19. This highlights the challenge of designing surveillance systems resilient to changes in testing policy and care-seeking behavior.

Our findings corroborate those of Loenenbach et al [32] on how SARS-CoV-2 incidence rates estimated using participatory surveillance were temporally correlated with wastewater testing indices but were higher than official national data, particularly after free testing ended. Both studies emphasize the limitations of case notifications and the importance of community and environmental surveillance. We additionally propose ways to incorporate alternative indicators of epidemic activity through regression modeling. More crucially, we also demonstrated how our estimates of both medically attended and unattended infections approximate serological data, thereby validating the approach. Doing so builds confidence in an alternative framework to accurately quantify attack rates in near real time. Near real-time point prevalence was previously estimated using household polymerase chain reaction survey platforms, such as England’s REACT [52] study, but with higher costs and complexity. A participatory surveillance cohort with limited diagnostic data from a subset of participants, augmented with routinely collected external indicators such as WVI [32], health care staff testing [53], or positivity rates from sentinel surveillance [54], would be more sustainable.

Validation against serological data should also be interpreted in light of evolving population immunity (Multimedia Appendix 1). Agreement between participatory surveillance–based estimates and seroconversion rates was strongest in earlier intervals, when most participants were serologically naïve, and incident infections largely represented primary infections. In the final interval of the serological cohort, a substantial proportion of participants were already seropositive at baseline, where we do not have validated methods for estimating the proportion infected, and it was effectively assumed that infections contributed by these individuals were negligible relative to infections in serologically naïve individuals. This likely explains why the incidence estimated on the basis of seroconversions alone was less than the incidence estimated from syndromic reports.

The strengths of this work include having a clearly defined denominator population, enabling true incidence-rate estimation compared to solely relying on other methods, such as clinic-based sentinels [54] and wastewater testing, both currently used in Singapore; the flexibility to integrate different indicators of community transmission; and triangulation with independent serological end points, lending validity to our approach.

The limitations include that 15% of illness reports fell outside the 28-day recall window, and recall bias may still exist within episodes. Symptom severity can influence testing decisions and health care access, potentially confounding results despite adjustments. Missing data on symptoms, episode dates, or test status were imputed, which might bias findings. Wastewater viral-load data are infrastructure-dependent and can be biased by virological [55] and climatological [56] factors. Due to the limited data points available, our validation of sentinel clinic test positivity as an alternative proxy indicator was less robust. The current method still needs a large, unbiased cohort with a subset willing to be tested and self-report results consistently. To replace unsustainable financial incentives, participatory surveillance could be scaled through citizen science approaches [57]. Alternative approaches could combine cohort symptom data with independent case-control study estimates [58] or sentinel clinic positivity rates stratified to match the symptom profiles of those in the participatory surveillance cohort. Also, the participatory surveillance cohort analyzed in this study was not designed to be population-representative, and participants were over-represented in some demographic areas. Recruitment pathways were also heterogeneous, including door-to-door enrollment, linkage from other cohorts, and participant referrals. Although cohort demographics differ from the national population, the resulting bias cannot be predetermined. Logistic regression (Table 3) showed no significant associations between demographic variables and COVID-19 diagnosis, even after adjustments; thus, demographic weighting was not used. Finally, behavioral responses and policies changed significantly during the study, including shifts in testing access and health care guidance, which may have influenced health care–seeking and testing across subgroups and affected estimates, especially in later epidemic phases. These factors likely contributed to the decline in medically attended cases across waves (Figure 2A or B), independent of changes in disease severity. These features may be associated with differences in health engagement, health care–seeking behavior, and testing behavior, although the direction and magnitude of any resulting bias cannot be quantified directly from the available data.

This study discusses integrating participatory surveillance into public health monitoring. These are adaptable, not fixed: (1) use wastewater data as a temporal independent signal of epidemic activity to supplement participatory data on illness episodes that estimate attack rates despite changes in incidence of infection, health-seeking, and testing. Public agencies should thus seek to integrate such wastewater reports with routine updates. (2) Focus on maintaining cohort participation and diagnostic consistency rather than merely increasing size. Systems should prioritize the retention of a small subpopulation with linkage to diagnostics and use this to periodically calibrate the parameters used to estimate infection and attack rates.

To conclude, we demonstrated how participatory syndromic surveillance, linked to a reliable epidemic proxy, offers timely, credible estimates of infection burden, complementing traditional and serological systems. By accounting for variability in health care–seeking and testing, it overcomes the limitations of passive surveillance and is timelier than serology. It can provide a scalable model for epidemic monitoring amid rapid behavioral and policy shifts.

## Supplementary material

10.2196/85608 Multimedia Appendix 1 Methods used to calculate incidence rates and ascertainment fractions, worked example to calculate weighting rules and bootstrap parameters.

10.2196/85608 Checklist 1 STROBE checklist.

## Abbreviations

ART: antigen rapid test
SOCRATEs: Strengthening Our Community’s Resilience Against Threats from Emerging Infections
STROBE: Strengthening the Reporting of Observational Studies in Epidemiology
WVI: wastewater viral-load index
