# Supplementary material for: Harnessing Participatory Surveillance Cohorts and Proxy Indicators to Dynamically Track Epidemic Trends and Undiagnosed COVID-19 Infections in Singapore: Longitudinal Observational Study
Source: J Med Internet Res. 2026 Jun 10;28:e85608. doi: 10.2196/85608 (PMC13252705; doi:10.2196/85608)
Supplement: Multimedia Appendix 1 [file jmir-v28-e85608-s001.docx]

Appendix 1. Mathematical formulation of methods used for calculation of incidence rates and ascertainment fractions, and sample size considerations

Overview of notation, unit of analysis and assignment of weights

In this description, we use small letters to denote variables for individual observations, and capital letters for variables derived by summation of individual observations over specified intervals (Table 1-1).

| **Table 1-1: Variables for individual observations and derived from summation of variables** | | | |
| --- | --- | --- | --- |
| **Individual observations** | | **Derived from summation of variables*** | |
| **Variable name** | **Nota-tion** | **Variable name** | **Nota-tion** |
| Participant identifier | $p$ | Sum of person-days at-risk | $D_{i}$ |
| Survey response identifier | $r$ | Sum of medically attended COVID-19 episodes | $C_{i}$ |
| Time (as calendar date) | $t$ | Sum of unattended self-diagnosed COVID-19 episodes | $S_{i}$ |
| participant–response–day  (unit of analysis) | p,r,t | Estimated probability of COVID-19 in ***unattended*** episodes | $F_{t}$ |
| Medically attended  (1 = yes, 0 = no) | $m_{p,r}$ | Estimated probability of COVID-19 in ***unattended*** episodes with ***NO*** *diagnosi*s | $G_{t}$ |
| Diagnosed with COVID-19  (1 = yes, 0 = no) | $c_{p,r}$ | Medically attended fraction, by estimating COVID-19 in unattended episodes | $G_{t}M_{i}$ |
| Valid for regression analyses based on symptoms (1 = yes, 0 = no) | $v_{p,r}$ | Medically attended fraction, by estimating COVID-19 in ***unattended*** episodes with ***NO*** diagnosis | $L_{i}$ |
| Date of survey response | $s_{p,r}$ |  | $L_{i}$ |
| Onset date | $o_{p,r}$ |  |  |
| Distribution of person-days at-risk | $d_{p,r,t}$ |  |  |
| Weight for illness episodes by time | $w_{p,r,t}$ |  |  |
| Estimated probability of COVID-19, unattended episodes | $\hat{f}_{p,r,t}$ |  |  |
| Estimated probability of COVID-19, undiagnosed episodes | $\hat{g}_{p,r,t}$ |  |  |
| *For these, $i$ denotes a specified interval which begins on date $b$ and ends on date $e$. | | | |

We organized our data so that the unit of analysis was participant-response-day (See Appendix 2), where each participant, $p$, could contribute one or more survey responses, $r$, with each response expanded to give up to 28 days of person-days over all the calendar dates, $t$, prior to the survey response date. This represents the lookback period of 4 weeks from the date of each survey response, $s$, for which respondents were asked to report illness and COVID-19 episodes.

For incidence rate calculations, person-days at-risk ($d_{p,r,t}$) is contributed by each participant on every occasion they provided a survey response regardless of whether they reported any episodes, so that:

$d_{p,r,t}=1$ when $t \leq s$ and $t>s-28$, whereas $d_{p,r,t}=0$ for all other values of $t$*.*

| **Table 1-2: Values for episode weights** $\boldsymbol{w}_{\boldsymbol{p,r,t}}$ **by days before survey response date,** $\boldsymbol{s}$ | | | | | | | | |
| --- | --- | --- | --- | --- | --- | --- | --- | --- |
|  | **Medically attended, no COVID-19 diagnosis** | | **Medically attended, diagnosed with COVID-19** | | **Unattended, No COVID-19 diagnosis** | | **Unattended, diagnosed with COVID-19** | |
|  | No. | % | No. | % | No. | % | No. | % |
| Total episodes | 876 | 100.0 | 577 | 100.0 | 747 | 100.0 | 271 | 100.0 |
| Had onset dates | 439 | 50.1 | 499 | 86.5 | 375 | 49.8 | 203 | 74.9 |
|  |  |  |  |  |  |  |  |  |
| Survey minus onset date ($s-o$) | No. | Weight | No. | Weight | No. | Weight | No. | Weight |
| 0 | 0 | 0.0000 | 5 | 0.01 | 6 | 0.0160 | 1 | 0.0049 |
| 1 | 5 | 0.0114 | 10 | 0.02 | 19 | 0.0507 | 6 | 0.0296 |
| 2 | 17 | 0.0387 | 15 | 0.0301 | 22 | 0.0587 | 6 | 0.0296 |
| 3 | 21 | 0.0478 | 24 | 0.0481 | 28 | 0.0747 | 9 | 0.0443 |
| 4 | 16 | 0.0364 | 15 | 0.0301 | 34 | 0.0907 | 7 | 0.0345 |
| 5 | 21 | 0.0478 | 26 | 0.0521 | 25 | 0.0667 | 7 | 0.0345 |
| 6 | 21 | 0.0478 | 10 | 0.02 | 24 | 0.0640 | 6 | 0.0296 |
| 7 | 20 | 0.0456 | 14 | 0.0281 | 12 | 0.0320 | 5 | 0.0246 |
| 8 | 14 | 0.0319 | 18 | 0.0361 | 11 | 0.0293 | 6 | 0.0296 |
| 9 | 24 | 0.0547 | 21 | 0.0421 | 18 | 0.0480 | 10 | 0.0493 |
| 10 | 16 | 0.0364 | 20 | 0.0401 | 15 | 0.0400 | 8 | 0.0394 |
| 11 | 25 | 0.0569 | 16 | 0.0321 | 12 | 0.0320 | 5 | 0.0246 |
| 12 | 16 | 0.0364 | 12 | 0.024 | 10 | 0.0267 | 9 | 0.0443 |
| 13 | 9 | 0.0205 | 14 | 0.0281 | 10 | 0.0267 | 7 | 0.0345 |
| 14 | 11 | 0.0251 | 13 | 0.0261 | 9 | 0.0240 | 4 | 0.0197 |
| 15 | 13 | 0.0296 | 14 | 0.0281 | 14 | 0.0373 | 10 | 0.0493 |
| 16 | 18 | 0.0410 | 7 | 0.014 | 9 | 0.0240 | 5 | 0.0246 |
| 17 | 14 | 0.0319 | 9 | 0.018 | 15 | 0.0400 | 10 | 0.0493 |
| 18 | 6 | 0.0137 | 13 | 0.0261 | 8 | 0.0213 | 7 | 0.0345 |
| 19 | 13 | 0.0296 | 19 | 0.0381 | 4 | 0.0107 | 8 | 0.0394 |
| 20 | 11 | 0.0251 | 16 | 0.0321 | 2 | 0.0053 | 7 | 0.0345 |
| 21 | 14 | 0.0319 | 12 | 0.024 | 4 | 0.0107 | 5 | 0.0246 |
| 22 | 9 | 0.0205 | 7 | 0.014 | 4 | 0.0107 | 2 | 0.0099 |
| 23 | 9 | 0.0205 | 10 | 0.02 | 7 | 0.0187 | 6 | 0.0296 |
| 24 | 10 | 0.0228 | 16 | 0.0321 | 4 | 0.0107 | 4 | 0.0197 |
| 25 | 14 | 0.0319 | 10 | 0.02 | 4 | 0.0107 | 5 | 0.0246 |
| 26 | 6 | 0.0137 | 17 | 0.0341 | 4 | 0.0107 | 3 | 0.0148 |
| 27 | 1 | 0.0023 | 11 | 0.022 | 11 | 0.0293 | 8 | 0.0394 |
| ≥28 | 65 | 0.0000 | 105 | 0.0000 | 30 | 0.0000 | 27 | 0.000 |
| Above was based on illness episodes where the respondent specified onset dates. The specified episode dates ranged from 15 Aug 2021 to 21 Nov 2022. Refer to Appendix 2 for worked examples. | | | | | | | | |

For survey responses reporting an illness episode, we weighted the observations as follows. For episodes with a specific onset date, $o$, we assigned a full weight of 1 for the corresponding date, i.e. $w_{p,r,t}=1$ when $t=o$, whereas $w_{p,r,t}=0$ for all other dates. We did likewise for 40 episodes where respondents did not fall sick but reported self-testing positive by Antigen Rapid Tests (ART) in the last 4 weeks. For these, we assumed the infection occurred in the midpoint of a 28-day window BEFORE their response, i.e. $o=s-14$. We excluded all reported episodes with specified onset dates more than 28 days BEFORE the survey date (i.e. when $s-o \geq28$ ), since our question asked specifically only for episodes within the last 28 days.

However, out of 2,471 illness episodes reported, onset dates were not specified in 955 (38.65%), with significant differences by COVID-19 diagnosis status and whether the episode was medically attended. For episodes without a specified onset date, we assumed the onset was likely distributed across the lookback period of 28 days as guided by the 1,516 episodes where specific onset dates were reported for the respective categories in Table 1-1. Note across the four categories, between 8% to 21% of specified onset dates were more than 28 days BEFORE the survey date. Our weighting method implicitly applies this same proportion

to those without a specified onset date, because $w_{p,r,t}=0$ for time points 28 days or later, with the sum of weights assigned to each episode being correspondingly less than 1.

Aggregating units of observation for presentation and analysis

For incidence rate calculations, the denominator, $D_{i}$, is the sum of person-days at-risk. For a given interval, $i$, with the start date of $b$ and the end date of $e$ this would be:

$$D_{i}=\sum_{t=b}^{e} w_{p,r,t}$$

Likewise, for medically attended COVID-19 episodes, $C_{i}$:

$$C_{i}=\sum_{t=b}^{e} \left[ w_{p,r,t}\times m\times c \right]$$

where $m=1$ and $m=0$ respectively denote episodes which were and were not medically attended, while $c=1$ and $c=0$ respectively denote episodes which were and were not diagnosed (by a doctor or by self) with COVID-19. Correspondingly, for self-diagnosed COVID-19 episodes ($S_{i}$):

$$S_{i}=\sum_{t=b}^{e} \left[ w_{p,r,t}\times\left( 1-m \right)\times c \right]$$

To aggregate observations for proportions and incidence rates by month (Figure 2), the start ($b$) and end ($e$) dates for each interval, $i$, are the first and last days of each calendar month in Table 1-3. The same notation was also used to derive the medically attended fraction for comparison with serological data (Figure 3), where the first and last day of the respective intervals are given Table 1-4.

Logistic regression models, predicted probabilities of COVID-19

During the study period, there was a policy stipulating that medically attended illness episodes with symptoms suggestive of COVID-19 should be tested accordingly. We hence assumed there would be little undiagnosed COVID-19 in medically attended episodes, and the reported COVID-19 status for these episodes would be reasonably accurate. We fitted multi-level logistic regression models restricted to these medically attended episodes, where the dependent variable was COVID-19 status. The multi-level structure had random intercept terms to represent 3 nested levels: days of observation derived from a given survey response, with up to several responses from each given participant. Independent variables were symptoms and proxy indicators of COVID-19 activity for the corresponding dates associated with the episodes, with demographic variables also included as potential confounders. Since the intent was to use symptoms to weight the possibility that an illness episode might be COVID-19, we fitted the regression model only to episodes where the respondent could recall at least 1 or more of the symptoms we included in our surveys, which were cough, runny nose, sore throat, breathlessness, anosmia and fever.

These fitted models were then used to predict the probability, $\hat{f}_{p,r,t}$, that each observation might be COVID-19, given the reported symptom profile and levels of the proxy indicators of COVID-19 activity corresponding to the dates when the episode likely occurred. Using $\hat{f}_{p,r,t}$, we can estimate the weighted probability that an observation would be COVID-19 for a given time point for observations which are not medically attended:

$$F_{t}={\sum_{p,r} \left[ w_{p,r,t}\times v_{p,r}\times\hat{f}_{p,r,t}\times\left( 1-m \right) \right]}/{\sum_{p,r} \left[ w_{p,r,t}\times v_{p,r}\times\left( 1-m \right) \right]}$$

where $v_{p,r}$ denotes whether the observation is valid (for regression or prediction using symptoms), $\sum_{p,r} \left[ w_{p,r,t}\times\left( 1-m \right)\times v \right]$ gives the total denominator of valid observations that are medically ***unattended***, and $\left[ w_{p,r,t}\times\hat{f}_{p,r,t}\times\left( 1-m \right)\times v \right]$ is the numerator, which additionally incorporates $\hat{f}_{p,r,t}$, the predicted probability of COVID-19 for that observation.

We can likewise apply the same previously fitted models to estimate the probability, $\hat{g}_{p,r,t}$, that unattended episodes with NO reported COVID-19 diagnosis might be COVID-19, where:

$$G_{t}={\sum_{p,r} \left[ w_{p,r,t}\times\hat{f}_{p,r,t}\times\left( 1-m \right)\times\left( 1-c \right)\times v \right]}/{\sum_{p,r} \left[ w_{p,r,t}\times\left( 1-m \right)\times\left( 1-c \right)\times v \right]}$$

The above restricts to only the episodes with NO COVID-19 diagnosis by adding the term $\left( 1-c \right)$ to our expression.

Deriving the medically attended fraction for different time periods

To estimate infections that were not medically attended, we used two different methods. Both assume that COVID-19 status is accurately determined for medically attended episodes, but that we need to estimate the true amount of COVID-19 in the medically unattended episodes.

Method 1 is applicable if the reported self-diagnosed episodes are either unreliable or unavailable. To estimate the number of COVID-19 infections, we thus multiply by $F_{t}$ all observations that were medically unattended regardless of COVID-19 diagnosis. This would include observations derived from episodes which did not report any data on symptoms, by assuming these followed the probability of being COVID-19 for observations with symptoms reported in the same period. The medically attended fraction, by estimating COVID-19 in all ***unattended*** episodes, would be:

$$M_{i}={C_{i}}/\left( C_{i}+\sum_{t=b}^{e} \left[ w_{p,r,t}\times F_{t}\times\left( 1-m \right) \right] \right)$$

where $\sum_{t=b}^{e} \left[ w_{p,r,t}\times F_{t}\times\left( 1-m \right) \right]$ gives the estimated contribution of COVID-19 from medically unattended episodes.

Method 2 applies if we assume reported self-diagnosed episodes are mostly accurate, so the remaining fraction of COVID-19 to be estimated is restricted to those medically unattended episodes which had NO COVID-19 diagnosis. Here we multiply by $G_{t}$ all observations that were medically unattended and had ***NO COVID-19 diagnosis***, while also adding the self-diagnosed episodes, $S_{i}$, as follows:

$$L_{i}={C_{i}}/\left( C_{i}+S_{i}+\sum_{t=b}^{e} \left[ w_{p,r,t}\times G_{t}\times\left( 1-m \right)\times\left( 1-c \right) \right] \right)$$

where $\sum_{t=b}^{e} \left[ w_{p,r,t}\times G_{t}\times\left( 1-m \right)\times\left( 1-c \right) \right]$ gives the estimated contribution of COVID-19 from medically unattended episodes with NO reported COVID-19 diagnosis.

Confidence intervals presented in Figure 3 were generated using the above formulas through a bootstrapping method with 1000 replicates implemented in Stata 18.0 (See Appendix 3).

Proxy indicators of COVID-19 epidemic activity

We postulated that the accuracy of estimates could be improved by including some proxy indicator of COVID-19 epidemic activity as an independent variable when fitting the regression models. We ran separate models with the following options for proxy indicators:

1. Incidence rate of COVID-19 based on medically attended episodes in cohort
2. Incidence rate of COVID-19 in TTSH healthcare staff reported in Sutjipto et al[42]
3. Average of Wastewater Viral-load Index (WVI) reported by the National Environment Agency[35]
4. Average of Sentinel clinic % positive reported by the Communicable Disease Agency[43]
5. No proxy indicators of COVID-19 activity included

| **Table 1-3: Indicators of COVID-19 epidemic activity** | | | | | | | | |
| --- | --- | --- | --- | --- | --- | --- | --- | --- |
| **Indi-cator** | **COVID-19 based on medically attended episodes in cohort** | | | | **2. Inci-dence per 1,000 health-care staff days** | **3. Waste-water Viral-load Index** | **4. Sentinel clinic % positive** | **Noti-fied cases per 1,000 per-son days** |
| **Year-month of analy-sis** | **No. of person days** | **Weigh-ted no. of epi-sodes** | **Weigh-ted no. are COVID-19** | **1. Inci-dence per 1,000 person days** |  |  |  |  |
| Aug’21 | 3335 | 1.565 | 0.000 | 0.003† | 0.020‡ | 7.70 | - | 0.016 |
| Sep’21 | 36307 | 64.182 | 1.000 | 0.028 | 0.205‡ | 31.31 | - | 0.177 |
| Oct’21 | 39780 | 74.720 | 16.000 | 0.402 | 0.661 | 153.63 | - | 0.602 |
| Nov’21 | 43188 | 71.486 | 13.760 | 0.319 | 0.457 | 94.96 | - | 0.406 |
| Dec’21 | 2839 | 3.675 | 0.030 | 0.011 | 0.103 | 27.94 | - | 0.087 |
| Jan’22 | 33499 | 69.000 | 16.866 | 0.503 | 1.123 | 128.15 | - | 0.392 |
| Feb’22 | 38668 | 144.204 | 93.605 | 2.421 | 5.529 | 653.38 | - | 2.434 |
| Mar’22 | 36884 | 110.325 | 72.557 | 1.967 | 4.584 | 536.14 | - | 2.200 |
| Apr’22 | 11980 | 31.703 | 14.657 | 1.223 | 1.617 | 140.55 | - | 0.620 |
| May’22 | 37006 | 118.017 | 45.794 | 1.237 | 1.813 | 115.89 | 33.68% | 0.623 |
| Jun’22 | 36478 | 105.453 | 47.335 | 1.298 | 2.770 | 195.46 | 34.35% | 0.860 |
| Jul’22 | 3632 | 11.642 | 7.455 | 2.053 | 4.577 | 474.94 | 50.32% | 1.543 |
| Aug’22 | 39440 | 105.541 | 34.277 | 0.869 | 1.913 | 197.87 | 31.09% | 0.740 |
| Sep’22 | 23360 | 60.459 | 17.673 | 0.757 | 1.207 | 112.36 | 15.89% | 0.420 |
| Oct’22 | 37763 | 164.022 | 56.259 | 1.49 | 3.490 | 387.11 | 30.60% | 1.150 |
| Nov’22 | 16645 | 65.890 | 18.321 | 1.101 | 1.350 | 102.20 | 16.63% | 0.401 |
| †Insufficient observations from cohort for month of Aug 2021; assigned to be 10% of values for Sep 2021 which approximates relative incidence of COVID-19 for case notifications in Singapore  ‡Published data available only from Oct 2021; value for Sep 2021 assigned to be 30% of values for Oct 2021, and Aug 2021 assigned to be 10% of Sep 2021 which approximates relative incidence of COVID-19 for case notifications in Singapore over these 3 successive months | | | | | | | | |

Data for indicator 3[42] was available only to the closest month, and we hence standardised our comparison by aggregating illness episodes for options 1 to 2 and averaging WVI data for option four at the level of year-months. However, our method would also work if data were available for temporal resolutions of weeks or days. Table 1-3 above shows the data used for options 1 to 5 for the period of analysis from August 2021 to November 2022. The values were log transformed as these more appropriately modelled periods with lower COVID-19 activity. For indicator 5, the data was only available from May 2022 onwards, but this was still included for some analyses given it is now part of our routine surveillance for COVID-19.

We also visualised our results against the incidence rate of COVID-19 case notifications in Singapore. [48] The daily number was aggregated by month and then divided by the population of Singapore in 2021 (5,453,566 persons) to obtain the average daily incidence rate per 1,000 person days shown in Table 1-3.

Comparison of results against medically attended fractions from serological study

We validated our results against independently derived estimates from a different approach for estimating the medically attended fraction. These came from the serological study found in[46] whose results are replicated in Table 1-4. As this serological study cohort differs from SOCRATES cohort (i.e. 2 separate distinct research studies), we cannot directly compare the data at the level of the individual participant and hence compared aggregated data over the intervals.

We excluded all participants who reported receiving “Sinovac” or “Sinopharm” vaccines at any time in the study (with most of having received their first dose before the earliest interval of interest to our study). This removes the interference from vaccine-induced antibodies, so that seroconversions in the remaining observations could be interpreted as serologically evidenced infection.

All blood samples for the serological study were tested at the National Public Health Laboratory using the Roche Elecsys® Anti-SARS-CoV-2 assay (Roche Diagnostics, Basel, Switzerland), designed for the detection of total antibodies (including IgG) directed against the nucleocapsid (N) antigen of SARS-CoV-2. Serum was separated by centrifugation and analysed on the Cobas e411 Immuno-analysers according to the manufacturer’s instructions. The assay employs a double-antigen sandwich format, with results expressed as a cut-off index (COI). A COI ≥1.0 was interpreted as reactive (antibody positive), and <1.0 as non-reactive (antibody negative), following the manufacturer’s guidelines. Internal quality controls and calibrators were included in each analytical run to ensure assay validity. Seroconversion was defined as a change from having a Roche Anti-N Cut-Off Index (COI) of <1 to ≥1 between two consecutive samples, with valid observations being intervals which were bookended by two consecutive samples.

While blood samples from the study were spread over several days for each study visit, we used the median date for each study visit to define the start and end points for the specified intervals. Intervals 1 to 3 correspond approximately to periods when the main circulating variants were Delta, Omicron BA.1/BA.2 and Omicron BA.5/XBB respectively.

| **Table 1-4: Serological attack rate and medically attended fraction from cohort study using Anti-N serology** | | | | | | | | | |
| --- | --- | --- | --- | --- | --- | --- | --- | --- | --- |
| **Main circulating variants in respective intervals†** | **Start of interval** | **End of interval** | **A. No. of valid ob-servations** | **B. No. Anti-N non-reac-tive at start** | **C. No. from A that sero-conver-ted** | **D. No. from C with medi-cally attended infections‡** | **E. % of A that sero-conver-ted**  **(C/A)** | **F. % of B that sero-conver-ted**  **(C/B)** | **G. % of C that was medi-cally att-ended (D/C)** |
| *1. Delta* | 23/8/21 | 13/1/22 | 1150 | 1145 | 56 | 33 | 4.9% | 4.9% | 58.9% |
| *2.BA.1/BA.2* | 14/1/22 | 27/6/22 | 994 | 944 | 408 | 252 | 41.0% | 43.2% | 61.8% |
| *3. BA.5/XBB* | 28/6/22 | 20/12/22 | 930 | 509 | 313 | 133 | 33.7% | 61.5% | 42.5% |
| Above table excludes individuals who received either the Sinovac or Sinopharm COVID-19 vaccines.  ‡Includes medically ascertained infections which were reported up to 14 days before the initial sample of the interval where seroconversions occurred.  †BA.1 and BA.2, BA.5 and XBB are sub-variants of omicron. | | | | | | | | | |

To estimate the proportion with medically attended infections from the serological study (column G), we included only participants who had a non-reactive result (i.e. COI < 1) on a Roche anti-N assay at the start of a given interval, and then subsequently became reactive (i.e. COI ≥ 1). The denominator was all such participants for a given interval (column C). The numerator was the subset of participants in that same interval who also reported being diagnosed with COVID-19 by a doctor (column D), including those where the diagnosis was up to 14 before the start of the interval, given the potential lag from symptom onset to seroconversion.

The above approach largely excludes those who were previously infected, since most of these would have had a reactive anti-N result in the earlier sample of the respective interval. However, this was a necessary limitation since there is no validated methodology for using paired serology to reliably detect re-infections in those who already had a reactive anti-N status in the earlier sample.

The results from column G of Table 1-4 were then used as a point of reference against which results for medically attended fractions from various estimation methods were compared. For each method and option for proxy indicators, we calculated the sum of the squares of the differences between the estimates and the result from serology for each interval. The best-performing combination was the one with the smallest sum of deviances across the three intervals.

In addition, we also compared the estimates of total infections from participatory surveillance against serology. Column E estimates the proportion infected by using seroconversions (column C) divided by all valid observations (column A). Since we cannot detect seroconversion in those already reactive at the start of the interval, this implicitly assumes that individuals who were already reactive on serology would be unlikely to get re-infected. Column F estimates the proportion infected by using seroconversions (column C) divided by only by those who were non-reactive at the start of that interval. If we apply this proportion also to individuals who were already reactive on serology, we would be assuming these individuals are just as likely to be infected as those who were non-reactive at the start of the interval. Both columns E and F would give similar cumulative population level incidence rates for intervals 1 (4.9% versus 4.9%) and 2 (41.0% versus 43.2%), since most of the population was uninfected at the start of those two intervals. However, for interval 3, when about half the population was already infected, assuming re-infections were reasonably rare (column E) would lead to incidence rates that are about half of what is estimated when using those who were serologically non-reactive at the start of the interval (see column F). Since those who were recently infected with omicron during the BA.1/BA.2 epidemic in interval 2 would be much less likely to be re-infected during the BA.5/XBB epidemics in interval 3, the true level of infections is likely closer to the estimate in column E (33.7%) than in column F (61.5%). We hence opted to compare estimates from the participatory surveillance cohort with the values from column E (see Figure 3B).

Sample size considerations

Recruitment of the cohort was done in several waves with the aim to recruit up to 2000 participants. This study uses all of the currently recruited participants from the SOCRATES cohort who responded to at least one survey wave between wave 28 and wave 38. No formal a priori sample size calculation was performed because analysis was based on an existing participatory surveillance cohort established before these study objectives were defined. The final sample consist of 1899 participants contributing 2284 episodes, including 756 COVID-19 episodes. This number of events was considered adequate for the analysis and estimation of incidence across epidemic waves.

**Appendix 2: Worked example on calculating weighting rule and unit of analysis (participant-response-day)**

For each survey response, we expanded observations to up to 28 person-days corresponding to the 28-day recall window preceding the response date. Each participant therefore contributes one observation per calendar day within this window, regardless of whether an illness episode was reported.

For illness episodes with a specified onset date within the 28-day window, the episode contributes a full weight of 1.0 to the corresponding onset day and 0 to all other days. Episodes with onset dates reported outside the 28-day window contribute zero weight and are excluded, consistent with the survey question wording (see below).

Repeated survey question to ask respondents if they fall sick in the past 28 days:

1. Did you fall sick at any time in the last 4 weeks?
   1. Yes, but I did not see a doctor
   2. Yes, and I saw a doctor at a clinic (GP clinic or polyclinic)
   3. Yes, and I saw a doctor at the hospital
   4. Yes, and I saw a doctor both at a clinic (GP clinic or polyclinic) and at the hospital
   5. Yes, and I saw a doctor via video consultation (e.g. telehealth platforms)
   6. No

For episodes without a specified onset date, we assigned fractional weights across the 28-day window. Specifically, the total episode weight was distributed across days according to the empirical distribution of onset timing (relative to survey date) observed among episodes of the same category (medically attended vs unattended, diagnosed vs undiagnosed) with known onset dates. This ensures that (i) the sum of weights for each episode is ≤1, and (ii) the implied timing of partially observed episodes mirrors that of fully observed episodes in the same stratum. Days ≥28 before the survey date receive zero weight, so episodes without onset dates may contribute a total weight <1, reflecting the probability that the true onset lay outside the recall window.

This is a simple worked example that walks through a single participant who submits one survey response, reports one illness episode without an onset date, and shows explicitly how the episode weight is apportioned across participant–response–days and how these weighted contributions enter both the numerator (episodes) and denominator (person-days) of incidence rate calculations.

| **Table 2-1**  **Working scenario 1**   - One participant (ID = P001) submits **one survey response** on survey date **Day 0**. - The illness episode recall window is in the **preceding 28 days** (Day −27 to Day 0). - The participant reports **one illness episode** that was:   - Medically **unattended**   - **Not** **diagnosed** with COVID-19   - **Onset date not specified**   Therefore, the episode is allocated fractionally across participant–response–days using **Table S2** weights $w_{p,r,t}$, where $t=$(survey date − onset date in days). Days with $t\geq28$receive weight 0 by definition (outside recall window). |
| --- |
| **A. Person-days at risk (denominator)**  For this single response, P001 contributes**:**   - 28 days × $1$person-day per day = **28 person-days** |
| **B. Weighted episode contribution (numerator)**  Weights (w) below are **copied directly from Table S2** (column: *Unattended, No COVID-19 diagnosis*).   \| **Survey minus onset date (**$\boldsymbol{s-o}$**)** \| **Episode weight (w)** \| \| --- \| --- \| \| 0 \| 0.0160 \| \| 1 \| 0.0507 \| \| 2 \| 0.0587 \| \| 3 \| 0.0747 \| \| 4 \| 0.0907 \| \| 5 \| 0.0667 \| \| 6 \| 0.0640 \| \| 7 \| 0.0320 \| \| 8 \| 0.0293 \| \| 9 \| 0.0480 \| \| 10 \| 0.0400 \| \| 11 \| 0.0320 \| \| 12 \| 0.0267 \| \| 13 \| 0.0267 \| \| 14 \| 0.0240 \| \| 15 \| 0.0373 \| \| 16 \| 0.0240 \| \| 17 \| 0.0400 \| \| 18 \| 0.0213 \| \| 19 \| 0.0107 \| \| 20 \| 0.0053 \| \| 21 \| 0.0107 \| \| 22 \| 0.0107 \| \| 23 \| 0.0187 \| \| 24 \| 0.0107 \| \| 25 \| 0.0107 \| \| 26 \| 0.0107 \| \| 27 \| 0.0293 \| \| **Sum** \| **0.9203** \| \| (t ≥ 28) \| 0.0000 \| |
| **C. Incidence rate contribution in this worked example**   - **Weighted episode count (numerator)** = $0.9203$ - **Person-days at risk (denominator)** = $28$   $\text{Incidence rate }=\text{ }\frac{0.9203}{28}\text{ }=\text{ }0.03287\text{ episodes per person-day}$  Optionally expressed per 1,000 person-days:  $0.03287\times1000=32.87\text{ episodes per 1,000 person-days}$ |
|  |
| **Working scenario 2**  **Scenario**   - Participant **P002** submits one survey response (**R1**) on survey date **Day 0**. - Recall window is **Day −27 to Day 0** (28 days). - Participant reports **one illness episode** with a **specified onset date**: onset occurred on **Day −5** (i.e., 5 days before the survey response). - Episode category (e.g., medically attended/unattended; COVID-19 diagnosis yes/no) is not material for the *onset-known* weighting rule—because the onset day is observed. |
| **A. Person-days at risk (denominator)**  For this single response, P001 contributes**:**   - 28 days × $1$person-day per day = **28 person-days** |
| **B. Weighted episode contribution (numerator)**  For **onset-known rule:**   - Assign **full weight 1.0** to the specific onset day and **0** to all other days in the recall window.   Here, onset is Day −5 ⇒ t$=5$.   \| **Survey minus onset date (**$\boldsymbol{s-o}$**)** \| **Episode weight (w)** \| \| --- \| --- \| \| 5 (corresponding to Day −5) \| **1.0000** \| \| all others \| 0.0000 \| \| **Sum over t=0 to 27** \| **1.0000** \| |
| **C. Incidence rate contribution in this worked example**   - **Weighted episode count (numerator)** = $1.0000$ - **Person-days at risk (denominator)** = $28$   $\text{Incidence rate }=\text{ }\frac{1.0000}{28}\text{ }=\text{ }0.03571\text{ episodes per person-day}$  Per 1,000 person-days:  $0.03571\times1000=35.71\text{ episodes per 1,000 person-days}$ |
|  |

**Appendix 3: Bootstrap method, parameters resampled and derivation of confidence intervals**

We used a participant-level bootstrap with 1,000 replicates, resampling participants with replacement and refitting the models in each replicate.

Uncertainty in the final incidence and medically attended fraction estimates was propagated using a bootstrap with 1,000 replicates, resampling participants with replacement and refitting the models in each replicate, and it was coded in Stata/SE 18 for Windows. For each bootstrap replicate:

1. Resampling unit and bootstrap procedure

The procedure involves two components to propagate uncertainty from the model and weighted episodes. First, resampling was done on observations to fit the logistic regression model, including the random effects structure as in the primary analysis. Secondly, resampling was performed at observations level that contributes to weighted illness episodes, such that the distribution of episode weights over participant-response-day units was re-derived for each replicate. This joint approach captures variability arising from model estimation and weighting of partially observed episodes.

1. Model refitting and prediction

For each replicate, the multilevel logistic regression model (with identical fixed effects, random intercept structure, and proxy indicator specification as in the primary analysis) was refitted using the resampled data. Predicted probabilities of COVID-19 for unattended episodes were then recomputed for that replicate.

1. Recalculation of estimates

Replicate-specific estimates of incidence rates, total infections, and medically attended fractions were derived by reapplying the same weighting and aggregation formulas to the replicate data.

1. Confidence interval construction

For each estimate, normal-based approximations for 95% confidence intervals was used.

**References**

1. Alvarez E, Bielska IA, Hopkins S, Belal AA, Goldstein DM, Slick J, Pavalagantharajah S, Wynfield A, Dakey S, Gedeon M-C, Alam E, Bouzanis K. Limitations of COVID-19 testing and case data for evidence-informed health policy and practice. Health Res Policy Syst 2023 Jan 25;21(1):11. doi: 10.1186/s12961-023-00963-1

2. Dimitris MC, Galea S, Marcus JL, Pan A, Sander B, Platt RW. What Has the Pandemic Revealed about the Shortcomings of Modern Epidemiology? What Can We Fix or Do Better? Am J Epidemiol 2022 May 20;191(6):980–986. doi: 10.1093/aje/kwac012

3. Zheng P, Li C, Zhang H, Huang B, Zhang Y, Feng H, Jiang D, Chen X, Dong X. Challenges of epidemiological investigation work in the COVID-19 pandemic: a qualitative study of the epidemiology workforce in Guangdong Province, China. BMJ Open British Medical Journal Publishing Group; 2022 Nov 1;12(11):e056067. PMID:36379656

4. Xu K, Gao B, Li J, Xiang Y, Cao L, Zhao M. Clinical features, diagnosis, and management of COVID-19 vaccine-associated Vogt-Koyanagi-Harada disease. Hum Vaccines Immunother Taylor & Francis; 2023 Aug 1;19(2):2220630. PMID:37282614

5. Lipsitch M, Riley S, Cauchemez S, Ghani AC, Ferguson NM. Managing and Reducing Uncertainty in an Emerging Influenza Pandemic. N Engl J Med Massachusetts Medical Society; 2009 July 9;361(2):112–115. doi: 10.1056/NEJMp0904380

6. WHO. Immunization Agenda 2030: A Global Strategy To Leave No One Behind. World Health Organization; 2020 Apr p. 60. Available from: https://www.who.int/publications/m/item/immunization-agenda-2030-a-global-strategy-to-leave-no-one-behind [accessed May 27, 2025]

7. Wynants L, Calster BV, Collins GS, Riley RD, Heinze G, Schuit E, Albu E, Arshi B, Bellou V, Bonten MMJ, Dahly DL, Damen JA, Debray TPA, Jong VMT de, Vos MD, Dhiman P, Ensor J, Gao S, Haller MC, Harhay MO, Henckaerts L, Heus P, Hoogland J, Hudda M, Jenniskens K, Kammer M, Kreuzberger N, Lohmann A, Levis B, Luijken K, Ma J, Martin GP, McLernon DJ, Navarro CLA, Reitsma JB, Sergeant JC, Shi C, Skoetz N, Smits LJM, Snell KIE, Sperrin M, Spijker R, Steyerberg EW, Takada T, Tzoulaki I, Kuijk SMJ van, Bussel BCT van, Horst ICC van der, Reeve K, Royen FS van, Verbakel JY, Wallisch C, Wilkinson J, Wolff R, Hooft L, Moons KGM, Smeden M van. Prediction models for diagnosis and prognosis of covid-19: systematic review and critical appraisal. BMJ British Medical Journal Publishing Group; 2020 Apr 7;369:m1328. PMID:32265220

8. Kalish H, Klumpp-Thomas C, Hunsberger S, Baus HA, Fay MP, Siripong N, Wang J, Hicks J, Mehalko J, Travers J, Drew M, Pauly K, Spathies J, Ngo T, Adusei KM, Karkanitsa M, Croker JA, Li Y, Graubard BI, Czajkowski L, Belliveau O, Chairez C, Snead KR, Frank P, Shunmugavel A, Han A, Giurgea LT, Rosas LA, Bean R, Athota R, Cervantes-Medina A, Gouzoulis M, Heffelfinger B, Valenti S, Caldararo R, Kolberg MM, Kelly A, Simon R, Shafiq S, Wall V, Reed S, Ford EW, Lokwani R, Denson J-P, Messing S, Michael SG, Gillette W, Kimberly RP, Reis SE, Hall MD, Esposito D, Memoli MJ, Sadtler K. Undiagnosed SARS-CoV-2 seropositivity during the first 6 months of the COVID-19 pandemic in the United States. Sci Transl Med American Association for the Advancement of Science; 2021 July 7;13(601):eabh3826. doi: 10.1126/scitranslmed.abh3826

9. Winter AK, Martinez ME, Cutts FT, Moss WJ, Ferrari MJ, McKee A, Lessler J, Hayford K, Wallinga J, Metcalf CJE. Benefits and Challenges in Using Seroprevalence Data to Inform Models for Measles and Rubella Elimination. J Infect Dis 2018 July 2;218(3):355–364. doi: 10.1093/infdis/jiy137

10. Lipsitch M, Santillana M. Enhancing Situational Awareness to Prevent Infectious Disease Outbreaks from Becoming Catastrophic. In: Inglesby TV, Adalja AA, editors. Glob Catastrophic Biol Risks Cham: Springer International Publishing; 2019. p. 59–74. doi: 10.1007/82_2019_172ISBN:978-3-030-36311-6

11. Guerrisi C, Turbelin C, Blanchon T, Hanslik T, Bonmarin I, Levy-Bruhl D, Perrotta D, Paolotti D, Smallenburg R, Koppeschaar C, Franco AO, Mexia R, Edmunds WJ, Sile B, Pebody R, van Straten E, Meloni S, Moreno Y, Duggan J, Kjelsø C, Colizza V. Participatory Syndromic Surveillance of Influenza in Europe. J Infect Dis 2016 Dec 1;214(suppl_4):S386–S392. PMID:28830105

12. Paolotti D, Carnahan A, Colizza V, Eames K, Edmunds J, Gomes G, Koppeschaar C, Rehn M, Smallenburg R, Turbelin C, Van Noort S, Vespignani A. Web-based participatory surveillance of infectious diseases: the Influenzanet participatory surveillance experience. Clin Microbiol Infect Off Publ Eur Soc Clin Microbiol Infect Dis 2014 Jan;20(1):17–21. PMID:24350723

13. Allen WE, Altae-Tran H, Briggs J, Jin X, McGee G, Shi A, Raghavan R, Kamariza M, Nova N, Pereta A, Danford C, Kamel A, Gothe P, Milam E, Aurambault J, Primke T, Li W, Inkenbrandt J, Huynh T, Chen E, Lee C, Croatto M, Bentley H, Lu W, Murray R, Travassos M, Coull BA, Openshaw J, Greene CS, Shalem O, King G, Probasco R, Cheng DR, Silbermann B, Zhang F, Lin X. Population-scale longitudinal mapping of COVID-19 symptoms, behaviour and testing. Nat Hum Behav Nature Publishing Group; 2020 Sept;4(9):972–982. doi: 10.1038/s41562-020-00944-2

14. Drew DA, Nguyen LH, Steves CJ, Menni C, Freydin M, Varsavsky T, Sudre CH, Cardoso MJ, Ourselin S, Wolf J, Spector TD, Chan AT, COPE Consortium. Rapid implementation of mobile technology for real-time epidemiology of COVID-19. Science American Association for the Advancement of Science; 2020 June 19;368(6497):1362–1367. doi: 10.1126/science.abc0473

15. Meta Research. Weights and Methodology Brief for the COVID-19 Symptom Survey by University of Maryland and Carnegie Mellon University, in Partnership with Facebook - Meta Research. Meta Res. Available from: https://research.facebook.com/publications/weights-and-methodology-brief-for-the-covid-19-symptom-survey-by-university-of-maryland-and-carnegie-mellon-university-in-partnership-with-facebook/ [accessed Aug 18, 2025]

16. Radin JM, Quer G, Pandit JA, Gadaleta M, Baca-Motes K, Ramos E, Coughlin E, Quartuccio K, Kheterpal V, Wolansky LM, Steinhubl SR, Topol EJ. Sensor-based surveillance for digitising real-time COVID-19 tracking in the USA (DETECT): a multivariable, population-based, modelling study. Lancet Digit Health Elsevier; 2022 Nov 1;4(11):e777–e786. PMID:36154810

17. Menni C, Valdes AM, Freidin MB, Sudre CH, Nguyen LH, Drew DA, Ganesh S, Varsavsky T, Cardoso MJ, El-Sayed Moustafa JS, Visconti A, Hysi P, Bowyer RCE, Mangino M, Falchi M, Wolf J, Ourselin S, Chan AT, Steves CJ, Spector TD. Real-time tracking of self-reported symptoms to predict potential COVID-19. Nat Med Nature Publishing Group; 2020 July;26(7):1037–1040. doi: 10.1038/s41591-020-0916-2

18. Rossman H, Keshet A, Shilo S, Gavrieli A, Bauman T, Cohen O, Shelly E, Balicer R, Geiger B, Dor Y, Segal E. A framework for identifying regional outbreak and spread of COVID-19 from one-minute population-wide surveys. Nat Med Nature Publishing Group; 2020 May;26(5):634–638. doi: 10.1038/s41591-020-0857-9

19. Atkins N, Harikar M, Duggan K, Zawiejska A, Vardhan V, Vokey L, Dozier M, de los Godos EF, Mcswiggan E, Mcquillan R, Theodoratou E, Shi T. What are the characteristics of participatory surveillance systems for influenza-like-illness? J Glob Health 13:04130. PMID:37856769

20. Greenleaf AR, Francis S, Zou J, Farley SM, Lekhela T, Asiimwe F, Chen Q. Influenza-Like Illness in Lesotho From July 2020 to July 2021: Population-Based Participatory Surveillance Results. JMIR Public Health Surveill 2024 Oct 8;10:e55208. PMID:39378443

21. Guerrisi C, Turbelin C, Souty C, Poletto C, Blanchon T, Hanslik T, Bonmarin I, Levy-Bruhl D, Colizza V. The potential value of crowdsourced surveillance systems in supplementing sentinel influenza networks: the case of France. Euro Surveill Bull Eur Sur Mal Transm Eur Commun Dis Bull 2018 June;23(25):1700337. PMID:29945696

22. Gertz A, Rader B, Sewalk K, Varrelman TJ, Smolinski M, Brownstein JS. Decreased Seasonal Influenza Rates Detected in a Crowdsourced Influenza-Like Illness Surveillance System During the COVID-19 Pandemic: Prospective Cohort Study. JMIR Public Health Surveill 2023 Dec 28;9:e40216. PMID:38153782

23. Adler AJ, Eames KTD, Funk S, Edmunds WJ. Incidence and risk factors for influenza-like-illness in the UK: online surveillance using Flusurvey. BMC Infect Dis 2014 May 1;14:232. PMID:24885043

24. Prieto JT, Jara JH, Alvis JP, Furlan LR, Murray CT, Garcia J, Benghozi P-J, Kaydos-Daniels SC. Will Participatory Syndromic Surveillance Work in Latin America? Piloting a Mobile Approach to Crowdsource Influenza-Like Illness Data in Guatemala. JMIR Public Health Surveill 2017 Nov 14;3(4):e87. PMID:29138128

25. Baltrusaitis K, Brownstein JS, Scarpino SV, Bakota E, Crawley AW, Conidi G, Gunn J, Gray J, Zink A, Santillana M. Comparison of crowd-sourced, electronic health records based, and traditional health-care based influenza-tracking systems at multiple spatial resolutions in the United States of America. BMC Infect Dis 2018 Aug 15;18(1):403. PMID:30111305

26. Kjelsø C, Galle M, Bang H, Ethelberg S, Krause TG. Influmeter - an online tool for self-reporting of influenza-like illness in Denmark. Infect Dis London, England; 2016 Apr;48(4):322–327. PMID:26654752

27. Carlson SJ, Dalton CB, Butler MT, Fejsa J, Elvidge E, Durrheim DN. Flutracking weekly online community survey of influenza-like illness annual report 2011 and 2012. Commun Dis Intell Q Rep 2013 Dec 31;37(4):E398-406. PMID:24882237

28. Richard A, Müller L, Wisniak A, Thiabaud A, Merle T, Dietrich D, Paolotti D, Jeannot E, Flahault A. Grippenet: A New Tool for the Monitoring, Risk-Factor and Vaccination Coverage Analysis of Influenza-Like Illness in Switzerland. Vaccines 2020 June 27;8(3):343. PMID:32605076

29. Rehn M, Carnahan A, Merk H, Kühlmann-Berenzon S, Galanis I, Linde A, Nyrén O. Evaluation of an Internet-based monitoring system for influenza-like illness in Sweden. PloS One 2014;9(5):e96740. PMID:24824806

30. Bajaj S, Chen S, Creswell R, Naidoo R, Tsui JL-H, Kolade O, Nicholson G, Lehmann B, Hay JA, Kraemer MUG, Aguas R, Donnelly CA, Fowler T, Hopkins S, Cantrell L, Dahal P, White LJ, Stepniewska K, Voysey M, Lambert B, Aguas R, Amswych M, Andersen-Waine B, Bajaj S, Bimpong K, Bodley A, Cantrell L, Chen S, Creswell R, Dahal P, Dickinson S, Dittrich S, Evans T, Ferguson-Lewis A, Franco C, Gao B, Hounsell R, Kasim M, Keene C, Lambert B, Mahmood U, Mills M, Moldokmatova A, Molyneux S, Naidoo R, Anye RN, Norman J, Pan-Ngum W, Pokharel S, Polner A, Rowe E, Saralamba S, Shretta R, Silal S, Stepniewska K, Tsui JL-H, Voysey M, Wanat M, White LJ. COVID-19 testing and reporting behaviours in England across different sociodemographic groups: a population-based study using testing data and data from community prevalence surveillance surveys. Lancet Digit Health Elsevier; 2024 Nov 1;6(11):e778–e790. PMID:39455191

31. Jiang L, Lee VJ, Lim WY, Chen MI, Chen Y, Tan L, Lin RT, Leo YS, Barr I, Cook AR. Performance of case definitions for influenza surveillance. Eurosurveillance European Centre for Disease Prevention and Control; 2015 June 4;20(22):21145. doi: 10.2807/1560-7917.ES2015.20.22.21145

32. Loenenbach A, Lehfeld A-S, Puetz P, Biere B, Abunijela S, Buda S, Diercke M, Dürrwald R, Greiner T, Haas W, Helmrich M, Prahm K, Schumacher J, Wedde M, Buchholz U. Participatory, Virologic, and Wastewater Surveillance Data to Assess Underestimation of COVID-19 Incidence, Germany, 2020–2024 - Volume 30, Number 9—September 2024 - Emerging Infectious Diseases journal - CDC. doi: 10.3201/eid3009.240640

33. Ahmed W, Simpson SL, Bertsch PM, Bibby K, Bivins A, Blackall LL, Bofill-Mas S, Bosch A, Brandão J, Choi PM, Ciesielski M, Donner E, D’Souza N, Farnleitner AH, Gerrity D, Gonzalez R, Griffith JF, Gyawali P, Haas CN, Hamilton KA, Hapuarachchi HC, Harwood VJ, Haque R, Jackson G, Khan SJ, Khan W, Kitajima M, Korajkic A, La Rosa G, Layton BA, Lipp E, McLellan SL, McMinn B, Medema G, Metcalfe S, Meijer WG, Mueller JF, Murphy H, Naughton CC, Noble RT, Payyappat S, Petterson S, Pitkänen T, Rajal VB, Reyneke B, Roman FA, Rose JB, Rusiñol M, Sadowsky MJ, Sala-Comorera L, Setoh YX, Sherchan SP, Sirikanchana K, Smith W, Steele JA, Sabburg R, Symonds EM, Thai P, Thomas KV, Tynan J, Toze S, Thompson J, Whiteley AS, Wong JCC, Sano D, Wuertz S, Xagoraraki I, Zhang Q, Zimmer-Faust AG, Shanks OC. Minimizing errors in RT-PCR detection and quantification of SARS-CoV-2 RNA for wastewater surveillance. Sci Total Environ 2022 Jan 20;805:149877. doi: 10.1016/j.scitotenv.2021.149877

34. McClary-Gutierrez JS, Aanderud ZT, Al-faliti M, Duvallet C, Gonzalez R, Guzman J, Holm RH, Jahne MA, Kantor RS, Katsivelis P, Kuhn KG, Langan LM, Mansfeldt C, McLellan SL, Grijalva LMM, Murnane KS, Naughton CC, Packman AI, Paraskevopoulos S, Radniecki TS, Roman FA, Shrestha A, Stadler LB, Steele JA, Swalla BM, Vikesland P, Wartell B, Wilusz CJ, Wong JCC, Boehm AB, Halden RU, Bibby K, Vela JD. Standardizing data reporting in the research community to enhance the utility of open data for SARS-CoV-2 wastewater surveillance. Environ Sci Water Res Technol The Royal Society of Chemistry; 2021 Aug 26;7(9):1545–1551. doi: 10.1039/D1EW00235J

35. NEA. Wastewater-based surveillance for COVID-19 and Zika. PUB; 2024. Available from: https://www.pub.gov.sg/-/media/PUB/Resources/Press-Releases/2024/04/LKYWP/For-website-upload-NEA-Media-Factsheet_Wastewater-Surveillance-FINAL.pdf [accessed May 27, 2025]

36. Wong JCC, Tan J, Lim YX, Arivalan S, Hapuarachchi HC, Mailepessov D, Griffiths J, Jayarajah P, Setoh YX, Tien WP, Low SL, Koo C, Yenamandra SP, Kong M, Lee VJM, Ng LC. Non-intrusive wastewater surveillance for monitoring of a residential building for COVID-19 cases. Sci Total Environ 2021 Sept 10;786:147419. doi: 10.1016/j.scitotenv.2021.147419

37. Lim VW, Lim RL, Tan YR, Soh AS, Tan MX, Othman NB, Borame Dickens S, Thein T-L, Lwin MO, Ong RT-H, Leo Y-S, Lee VJ, Chen MI. Government trust, perceptions of COVID-19 and behaviour change: cohort surveys, Singapore. Bull World Health Organ 2021 Feb 1;99(2):92–101. PMID:33551503

38. Ministry of Health. ANTIGEN RAPID TEST KITS VENDING MACHINES DEPLOYED ISLANDWIDE AND UPDATES TO HOME RECOVERY AND TRAVEL CLASSIFICATIONS. Minist Health. 2021. Available from: https://www.moh.gov.sg/newsroom/antigen-rapid-test-kits-vending-machines-deployed-islandwide-and-updates-to-home-recovery-and-travel-classifications_17sep2021/ [accessed Jan 23, 2025]

39. Ministry of Health. RESUMING OUR TRANSITION TOWARDS COVID RESILIENCE. Minist Health. 2021. Available from: https://www.moh.gov.sg/newsroom/resuming-our-transition-towards-covid-resilience/ [accessed Feb 27, 2025]

40. Muench P, Jochum S, Wenderoth V, Ofenloch-Haehnle B, Hombach M, Strobl M, Sadlowski H, Sachse C, Torriani G, Eckerle I, Riedel A. Development and Validation of the Elecsys Anti-SARS-CoV-2 Immunoassay as a Highly Specific Tool for Determining Past Exposure to SARS-CoV-2. J Clin Microbiol American Society for Microbiology; 2020 Sept 22;58(10):10.1128/jcm.01694-20. doi: 10.1128/jcm.01694-20

41. Lou B, Li T-D, Zheng S-F, Su Y-Y, Li Z-Y, Liu W, Yu F, Ge S-X, Zou Q-D, Yuan Q, Lin S, Hong C-M, Yao X-Y, Zhang X-J, Wu D-H, Zhou G-L, Hou W-H, Li T-T, Zhang Y-L, Zhang S-Y, Fan J, Zhang J, Xia N-S, Chen Y. Serology characteristics of SARS-CoV-2 infection after exposure and post-symptom onset. Eur Respir J European Respiratory Society; 2020 Aug 27;56(2). PMID:32430429

42. Sutjipto S, Aung AH, Soon MML, Jing C, Ang BSP, Sadarangani SP, Chong KW, Ng OT, Marimuthu K, Lim WY, Chow A, Vasoo S. Plastic Waste and COVID-19 Incidence Among Hospital Staff After Deescalation in PPE Use. JAMA Netw Open 2025 Apr 15;8(4):e255264. doi: 10.1001/jamanetworkopen.2025.5264

43. Mah T, Chae S-R, Wang L, Cheng S, Goh AXC, Khamis NH, Ho C, Keng B, Khong WX, Chen MI-C, Pung R, Cui L, Ma W, Chen B, Yeo B, Said Z, Lee VJ, Ho ZJM. Evaluating Acute Respiratory Infection and Influenza-Like Illness case definitions for community COVID-19 and influenza surveillance. Proceedings of the 12th edition of Options for the Control of Influenza; 2024 Sep 29; Brisbane, Australia. United Kingdom: International Society for Influenza and other Respiratory Virus Diseases; 2024.

44. MOH. Enablers to Support Safe Re-Opening. Minist Health. 2020. Available from: https://www.moh.gov.sg/newsroom/enablers-to-support-safe-re-opening/ [accessed May 27, 2025]

45. MOH. FURTHER RATIONALISATION OF SMMs, BOOSTING VACCINATIONS, AND UPDATES TO COVID-19 SUBSIDIES. Minist Health. 2022. Available from: https://www.moh.gov.sg/newsroom/further-rationalisation-of-smms-boosting-vaccinations-and-updates-to-covid-19-subsidies/ [accessed May 27, 2025]

46. Soh AS, Ong BWL, Chen MI. Longitudinal serological assessment of SARS-CoV-2 infection and medical ascertainment in a community cohort in Singapore between Year 2020–2023. Proceedings of the 19th Singapore Public Health & Occupational Medicine Conference 2025; 2025 Oct 23; Singapore, Singapore. Singapore: College of Public Health and Occupational Physicians; 2025.

47. Vandenbroucke JP, von Elm E, Altman DG, Gøtzsche PC, Mulrow CD, Pocock SJ, Poole C, Schlesselman JJ, Egger M. Strengthening the Reporting of Observational Studies in Epidemiology (STROBE): Explanation and elaboration. Int J Surg 2014 Dec 1;12(12):1500–1524. doi: 10.1016/j.ijsu.2014.07.014

48. World Health Organization. Global COVID-19 Overview | WHO COVID-19 dashboard. WHO Coronavirus COVID-19 Dashboard More Resour Dashboard. 2023. Available from: http://data.who.int/dashboards/covid19/summary [accessed Aug 15, 2025]

49. Lwin MO, Lu J, Sheldenkar A, Panchapakesan C, Tan Y-R, Yap P, Chen MI, Chow VT, Thoon KC, Yung CF, Ang LW, Ang BS. Effectiveness of a Mobile-Based Influenza-Like Illness Surveillance System (FluMob) Among Health Care Workers: Longitudinal Study. JMIR MHealth UHealth JMIR Publications Inc., Toronto, Canada; 2020 Dec 7;8(12):e19712. doi: 10.2196/19712

50. Lwin MO, Jayasundar K, Sheldenkar A, Wijayamuni R, Wimalaratne P, Ernst KC, Foo S. Lessons From the Implementation of Mo-Buzz, a Mobile Pandemic Surveillance System for Dengue. JMIR Public Health Surveill JMIR Publications Inc., Toronto, Canada; 2017 Oct 2;3(4):e7376. doi: 10.2196/publichealth.7376

51. Lwin MO, Yung CF, Yap P, Jayasundar K, Sheldenkar A, Subasinghe K, Foo S, Jayasinghe UG, Xu H, Chai SC, Kurlye A, Chen J, Ang BSP. FluMob: Enabling Surveillance of Acute Respiratory Infections in Health-care Workers via Mobile Phones. Front Public Health Frontiers; 2017 Mar 17;5. doi: 10.3389/fpubh.2017.00049

52. Riley S, Atchison C, Ashby D, Donnelly CA, Barclay W, Cooke GS, Ward H, Darzi A, Elliott P. REal-time Assessment of Community Transmission (REACT) of SARS-CoV-2 virus: Study protocol. Wellcome Open Res 2021 Apr 21;5:200. PMID:33997297

53. Venkatachalam I, Conceicao EP, Aung MK, How MKB, Wee LE, Sim JXY, Tan BH, Ling ML. Healthcare workers as a sentinel surveillance population in the early phase of the COVID-19 pandemic. Singapore Med J 2022 Oct;63(10):577. doi: 10.11622/smedj.2021083

54. Tan CY, Zeng K, Cui L, Lin RTP, Chen M. Diagnostic performance of rapid antigen tests (RAT) for COVID-19 and factors associated with RAT-negative results among RT-PCR-positive individuals during Omicron BA.2, BA.5 and XBB.1 predominance. BMC Infect Dis 2024 May 21;24(1):504. doi: 10.1186/s12879-024-09408-8

55. Prasek SM, Pepper IL, Innes GK, Slinski S, Betancourt WQ, Foster AR, Yaglom HD, Porter WT, Engelthaler DM, Schmitz BW. Variant-specific SARS-CoV-2 shedding rates in wastewater. Sci Total Environ 2023 Jan 20;857:159165. doi: 10.1016/j.scitotenv.2022.159165

56. Bitter LC, Kibbee R, Garant T, Örmeci B. Impact of wastewater characteristics and weather events on the N2 and N1 gene target ratios during wastewater surveillance of SARS-CoV-2 at five treatment plants and an upper sewershed location. Sci Total Environ 2025 June 15;981:179592. doi: 10.1016/j.scitotenv.2025.179592

57. Tan YR, Agrawal A, Matsoso MP, Katz R, Davis SLM, Winkler AS, Huber A, Joshi A, El-Mohandes A, Mellado B, Mubaira CA, Canlas FC, Asiki G, Khosa H, Lazarus JV, Choisy M, Recamonde-Mendoza M, Keiser O, Okwen P, English R, Stinckwich S, Kiwuwa-Muyingo S, Kutadza T, Sethi T, Mathaha T, Nguyen VK, Gill A, Yap P. A call for citizen science in pandemic preparedness and response: beyond data collection. BMJ Glob Health BMJ Publishing Group Ltd; 2022 June 27;7(6). PMID:10.1136/bmjgh-2022-009389

58. Mao Y, Tan Y-R, Thein TL, Chai YAL, Cook AR, Dickens BL, Lew YJ, Lim FS, Lim JT, Sun Y, Sundaram M, Soh A, Tan GSE, Wong FPG, Young B, Zeng K, Chen M, Ong DLS. Identifying COVID-19 cases in outpatient settings. Epidemiol Infect 2021 Apr 5;149:e92. PMID:33814027
